# Incorporation of Functional Lung Imaging Into Radiation Therapy Planning in Patients With Lung Cancer: A Systematic Review and Meta-Analysis

**DOI:** 10.1016/j.ijrobp.2024.04.001

**Published:** 2024-04-15

**Authors:** Julie Midroni, Rohan Salunkhe, Zhihui Liu, Ronald Chow, Gabriel Boldt, David Palma, Douglas Hoover, Yevgeniy Vinogradskiy, Srinivas Raman

**Affiliations:** * Temerty Faculty of Medicine, University of Toronto, Toronto, Canada; † Radiation Medicine Program, Princess Margaret Cancer Center, Toronto, Canada; ‡ Department of Radiation Oncology, University of Toronto, Toronto, Canada; § Biostatistics, Princess Margaret Cancer Center, Toronto, Canada; ‖ London Regional Cancer Program, London Health Sciences Centre, Schulich School of Medicine and Dentistry, University of Western Ontario, London, Canada; ¶ Ontario Institute for Cancer Research, Toronto, Canada; # Department of Radiation Oncology, University of Colorado School of Medicine, Aurora, United States of America; ** Department of Radiation Oncology, Thomas Jefferson University, Philadelphia, United States of America

## Abstract

Our purpose was to provide an understanding of current functional lung imaging (FLI) techniques and their potential to improve dosimetry and outcomes for patients with lung cancer receiving radiation therapy (RT). Excerpta Medica dataBASE (EMBASE), PubMed, and Cochrane Library were searched from 1990 until April 2023. Articles were included if they reported on FLI in one of: techniques, incorporation into RT planning for lung cancer, or quantification of RT-related outcomes for patients with lung cancer. Studies involving all RT modalities, including stereotactic body RT and particle therapy, were included. Meta-analyses were conducted to investigate differences in dose-function parameters between anatomic and functional RT planning techniques, as well as to investigate correlations of dose-function parameters with grade 2+ radiation pneumonitis (RP). One hundred seventy-eight studies were included in the narrative synthesis. We report on FLI modalities, dose-response quantification, functional lung (FL) definitions, FL avoidance techniques, and correlations between FL irradiation and toxicity. Meta-analysis results show that FL avoidance planning gives statistically significant absolute reductions of 3.22% to the fraction of well-ventilated lung receiving 20 Gy or more, 3.52% to the fraction of well-perfused lung receiving 20 Gy or more, 1.3 Gy to the mean dose to the well-ventilated lung, and 2.41 Gy to the mean dose to the well-perfused lung. Increases in the threshold value for defining FL are associated with decreases in functional parameters. For intensity modulated RT and volumetric modulated arc therapy, avoidance planning results in a 13% rate of grade 2+ RP, which is reduced compared with results from conventional planning cohorts. A trend of increased predictive ability for grade 2+ RP was seen in models using FL information but was not statistically significant. FLI shows promise as a method to spare FL during thoracic RT, but interventional trials related to FL avoidance planning are sparse. Such trials are critical to understanding the effect of FL avoidance planning on toxicity reduction and patient outcomes.

## Introduction

Lung cancer is the leading cause of cancer mortality globally, with a 5-year relative survival rate of 10% to 20%.^1^ Radiation therapy (RT) is a cornerstone of management in localized lung cancer.^2^ Radiation-induced lung toxicities (RILT) are common side effects caused by damage to healthy lung tissue and can result in morbidity, reduced quality of life, and compromised oncological outcomes.^3^

**Fig. 1.**
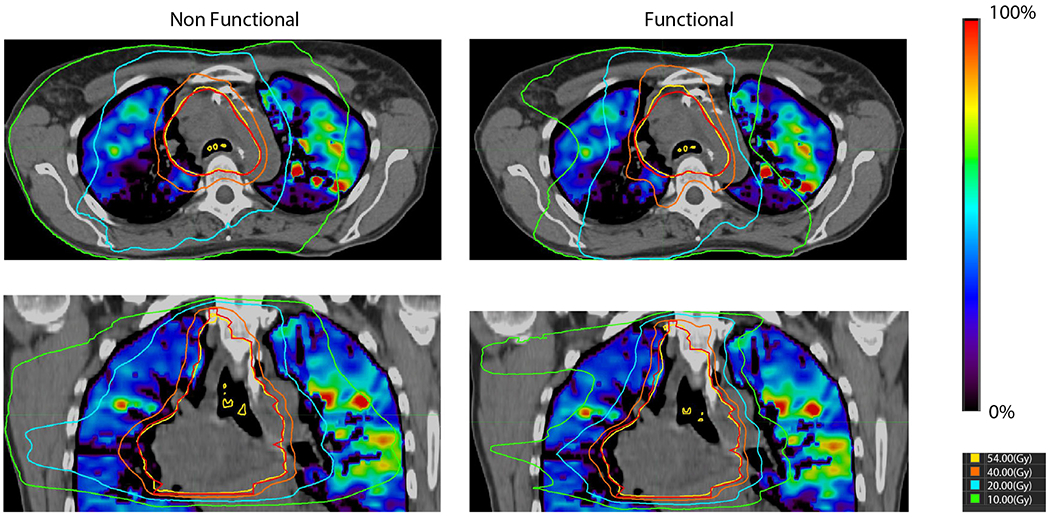
Example anatomic and functional avoidance radiation therapy plans. The functional plan demonstrates decreased irradiation of the high-functioning areas in the left lung.

Functional lung imaging (FLI) is an umbrella term for lung imaging modalities that characterize regions of lung that contribute to lung function – usually, ventilation (V) or perfusion (Q). These modalities highlight regions of functional importance to gas exchange.^4^ There has been growing interest in the use of FLI to enhance RT treatment planning.^4^ An example of one such plan is seen in Figure 1, wherein, compared with a standard plan, the FLI-guided plan spares regions of higher function in the left lung. The logic behind incorporating FLI into RT planning is to prioritize protection of lung tissues preferentially involved in gas exchange, potentially leading to reduced toxicity and improved functional outcomes.^4^ Unfortunately, most FLI modalities necessitate additional imaging, which can pose a substantial cost barrier and requires the synthesis of multiple imaging types, which can be challenging. Furthermore, at the moment, a lack of exploration into the optimal modality of FLI, as well as inconsistency between attempts to implement FLI in clinical RT planning, mean that the benefit of FLI to patient outcomes is not yet well-elucidated. There is thus a strong incentive for an in-depth exploration of the potential benefit FLI may have to determine whether it is a worthwhile addition to the care of patients with lung cancer.

There have been many studies exploring best methods for FLI, incorporating FLI into RT planning and correlating FLI-based parameters with RILT. Such studies use varying methods, definitions of functional lung (FL), and RT delivery modalities, with significant variability in techniques and results. The last review article to synthesize these data, by Bucknell et al,^4^ was published in 2018. Since then, many new studies on FLI and RT have been published, including a substantial increase in observational data and the advent of prospective clinical trial data. This warrants an in-depth, updated examination of FLI for treatment-planning, including novel analyses and the integration of new data into previous analyses.

This review article has 2 main objectives: assess improvements in FL irradiation for plans that incorporate FLI compared with plans that do not and assess correlations between FL irradiation and RILT. Secondarily, this article reports on FLI modalities, definitions of FLI, and dose-response relationships between irradiated FL and measures of lung function.

## Methods and Materials

### Literature review

The review is in concordance with Preferred Reporting Items for Systematic Reviews and Meta-Analyses (PRISMA) guidelines and was registered on the International Prospective Register of Systematic Reviews (PROSPERO) (CRD42022349032). PubMed, Excerpta Medica dataBASE (EMBASE), and Cochrane Library were searched from 1990 to April 2023, in English. The full protocol for the search strategy and screening criteria are available in supplementary material E1.

Title and abstract screening were conducted in-duplicate and independently by 2 review authors (JM and RS). Studies were included if they mentioned FLI modalities in any capacity related to adult lung cancer. Studies that reported on multiple types of cancer to the thorax, including lung cancer, were kept. Conflicts were resolved by a senior author (SR). At the full-text stage, the 2 reviewers screened the studies. Inclusion criteria were any paper detailing FLI methods for imaging patients with lung cancer treated with RT; FLI to inform RT planning for lung cancer; FLI-based quantification of change in lung function or development of toxicity due to RT in lung cancer; and using FLI to predict or correlate with any RILT in patients with lung cancer. Exclusion criteria were protocols, reviews, abstracts, or conference presentations; failure to report in the context of lung cancer; pediatric population related to anything other than technical details of FLI modalities; and regional perfusion quantification not related to identifying functional lung. Conflicts were again resolved by SR. Reference lists in included articles were then screened for additional sources. For all statistical analyses, risk of bias assessment was conducted in the form of the appropriate JBI checklist.

### Data collection

Data were collected twice in a predefined collection form, in-duplicate, and cross-checked. Items of note included FLI modality, study type, study population descriptors and size, FL definition, and any relevant outcome related to the study objective. Detailed headers for data extraction can be found in E2.

### Functional avoidance planning: Meta-analysis, sensitivity, and metaregression

The effect of RT on FL is assessed via dose-function metrics, which measure the dose of radiation to FL specifically. A meta-analysis was conducted to compare the mean difference of dose-functional parameters between functional plans, which minimize dose to FL, and anatomic (standard) plans. Included studies reported the unadjusted parameters of interest or patient data from which we calculated the parameters required. For studies that reported the relevant parameters individually for functional and anatomic plans, we used the provided *P* values and knowledge of the significance test used to impute the information. If not provided, we read numerical results from figures. If this was not possible, for studies where only medians and ranges/IQRs were available, we used previously established methods^5,6^ to calculate the required information. Barring these, we contacted corresponding authors for appropriate summary statistics. Some studies reported multiple outcomes for different RT modalities and definitions of FL. In these cases, we chose to include the best result (lowest *P* value, followed by greatest difference), while maintaining consistency between different techniques for the same study: if we chose 1 modality or definition for 1 parameter, we would use the same one for all other parameters from that study. Studies were excluded if they did not use V- or Q-imaging or if they failed to provide sufficient data to calculate dose-function parameters via one of the previously mentioned methods. Studies were also excluded if they had identical or sufficiently similar patient populations to larger studies. Papers where entire data sets of plans were deemed clinically unacceptable or had average planning target volume (PTV) under 80% were discarded. When corresponding data were provided, individual cases with PTV coverage under 80% were deemed clinically unacceptable and were excluded.

To assess the effect of the choice of specific FL definitions on functional dosimetry, we performed a sensitivity analysis and metaregression on eligible studies in the meta-analysis. First, we repeated the meta-analysis, swapping out definitions of FL for studies that reported the same parameters using multiple different definitions, and compared the results of the altered and original analyses. To further elucidate the influence these definitions have, we then took only studies that optimized their RT plans using a specific, thresholded definition of FL, and categorized them as high or low threshold using a predefined cutoff of 30% or 30th percentile and 70% or 70th percentile. The intermediate range was excluded to account for the difference between percentile and percentage threshold definitions. We performed metaregression between these 2 categories, to see if there were significant differences in dose-function parameters between the 2 groups.

### Radiation pneumonitis: Meta-analyses

We first assessed the predictive power of dose-function parameters to the incidence of grade 2+ radiation pneumonitis (RP) by comparing the ability of models to predict grade 2+ RP between those that used only anatomic information and those that incorporated functional information (V or Q separately). We did this through the area under the receiver operating characteristic curve (AUC) statistic for prediction of grade 2+ RP. Studies were included if they assessed the predictive ability of a model that predicted only grade 2+ RP. Studies that reported more broadly on other grades of RP, or which did not provide enough statistical information, were excluded. For studies that reported multiple models, we selected the best model available in each category. We then meta-analyzed the 3 groups of AUC values: anatomic, V, and Q, to determine whether functional information enhanced prediction of grade 2+ RP.

Finally, we collated all studies that used interventional avoidance planning and reported their rate of grade 2+ RP and performed a meta-analysis of the rate of grade 2+ RP. The details of this and all aforementioned analyses can be found in E3.

## Results

Of the 2550 studies that were screened, 178 were deemed relevant to the review. The detailed breakdown of screening can be seen in Figure EA.

### FLI modalities

There are many FLI modalities, which vary widely in technique and information obtained. Sixty-one papers described in detail, assessed performance of, or compared different modalities of FLI:

1. Single-photon emission computed tomography (SPECT): V or Q. Sixteen studies reported for perfusion^7–22^ and 14 for ventilation.^9,10,13,17,22–31^
2. Computed tomography (CT): referring to approaches to derive FL images from CT scans. The two main groups of methods for V are Jacobian-based^10,13,23–26,28,29,30,32–42,43,44,45^ (23 studies) and Hounsfield unit (HU)-based^10,12,24–26,28–30,32,34,35,38,40,41,46,43,47–49^ (19 studies). Twenty other methods were also reported for V.^24–28,33,39,41,43,44,49,45,50–57^ Even within their subcategories, CT-V methods are heterogeneous: the studies listed use a variety of techniques and forms of image registration, resulting in different performance characteristics.^10,23–26,28–30,34–36,38,40,41,43,45,52^ Q CT was less common, with 5 studies reporting in total.^11,14,15,16,20^
3. Positron emission tomography (PET): V or Q: 6 studies reported for Q^58–63^ and 10 studies reported for V.^34,38,40,55,58–61,62,64^ All studies, V and Q, used ^68^Ga-PET.
4. Magnetic resonance imaging (MRI): all ventilation, using either helium or xenon (Xe). Five studies were found.^43,46,65–67^
5. Dual energy CT (DECT): perfusion only. Three studies were found.^7,11,63^
6. Seven studies reported on FLI methods that were not V or Q based.^21,24,31,47,52,64,68,69^ These consisted of planar imaging,^47^ various surrogates for identifying diseased regions of lung,^64,68,69^ incorporating tumor anatomy,^21,31^ and stress mapping.^24,52^

Table E1 details all comparisons of other FLI modalities to SPECT. Given that CTs are obtained as standard of care for diagnostics and RT planning, there appears to be substantial interest in development of robust, CT-derived FLI methods. Nine papers in Table EA compared various CT-V methods derived from routine CT scans to SPECT V.^10,13,24–26,28–30,36^ The performance of Jacobian, HU, and other methods is variable, with Spearman coefficients (r_s_) as high as 0.82^23^ and as low as −0.02.^10^ No 1 method was consistently superior compared with the others, and no 1 method was consistent in Dice similarity coefficient or r_s_. Four studies compared CT-V with SPECT Q,^8,10,12,13^ with all correlations and comparative statistics found to be significant. One study reported on Xe-CT versus SPECT V and found that only 3 of 11 patients had a significant correlation to the SPECT V scan.^27^ A more general 2019 review was published on the subject of CT-V imaging, which contains CT-derived FLI compared with all other FLI modalities, with similar, variable results.^70^ CT-Q was less frequently studied, with only 4 (Table E1) papers comparing it to SPECT-Q.^14–16,58^ All correlations were significant and as low as 0.57.^58^ The other modality compared with SPECT was DECT.^7,11^ Although only 2 papers compared the efficacy of these methods, both found strong correlations (r ≥ 0.89).^7,11^

Interestingly, although both V and Q SPECT appear to be the standard for FLI in patients with thoracic cancer, Forghani et al^9^ found only modest correlations between them. They suggest that this discrepancy may be because of a small sample size, which prevented their comparisons from accounting for a wide variety of functional defects.^9^ Complementing this, Yuan et al^22^ found that 39% of patients had sufficient differences between V and Q SPECT scans, and that they would benefit from incorporating both into functional avoidance plans. Nakajima et al^12^ similarly found that radiation doses to ventilated regions were consistently and significantly higher than to perfused regions, indicating that they do not occupy identical space.

### Defining FL

In total, 95 studies defined FL via a threshold: using SPECT counts, image intensity, or some other metric, they determined a cutoff value of V or Q, above which tissue was considered to be functional.^8–11,13–16,18,19,21,24–26,28–30,32,34,39–42,45,50,53,55,57–60,62,66,69,71–131^ Thirteen studies had a physician or software annotate FL contours.^7,17,22,46,48,83,131–137^ Five studies used all lung tissue that was positive on the scan.^54,67,126,138,139^ Thirty-four studies used a weighting method^7,12,31,44,50,56,73,78,81,83,90,96,97,99,104,108,117,118,121,123,126,127,129,140–150^: instead of using a threshold or contour, the entire lung was weighted by the image intensity in each pixel. As such, there are no functional and nonfunctional lung classifications, but rather each voxel has a unique level of function relative to the others. This is a substantially different definition of FL compared with the thresholding method. Seven studies defined FL in a manner unrelated to V or Q.^39,64,68,69,126,128,151^ A table of definitions used in each study is included in Table E2.

Supporting data on optimal choice of FL are sparse. A comparison between threshold and weighted methods revealed superior AUC and odds ratios (significance not provided) for parameters derived using the thresholding method,^72^ in the context of predicting RP. Within thresholding methods, a theme that emerged was the correlation of certain thresholds with RILT development: Farr et al^87^ chose a 40th percentile threshold for this reason, and Ieko et al^93^ chose a 20th percentile by the same logic, as did Ding et al.^73^ A lower-end threshold was not, however, always the most predictive. Dhami et al^84^ found that a 70th-percentile threshold was the most predictive for grade 2+ RP. Similarly, Faught et al^90^ found that their best predictive models used a threshold at the 69th percentile, though they also showed minimal variation between AUC values for different thresholds.

Regarding robustness, although a lack of data for non-CT-based methods prevented us from analyzing the robustness of thresholding in various FLI modalities, it is worth noting that Siva et al^112^ found only a marginal difference in dose-function parameters when thresholding ventilated lung at 70% rather than 50% of the maximum value. For perfusion, Shioyama et al^110^ and Farr et al^87^ both found that for SPECT Q, changing the minimum cutoff value for defining a lung as functional still allowed for a significant improvement to dose-functional parameters with functional planning. The same was found by Lucia et al^116^ for Ga-PET Q. None of these studies aside from Siva et al performed a direct comparison between thresholds.

### Dose-response relationships

Generally, it was found that FL distribution changes after irradiation. Several studies explored changes in Q^59,72,74,76,82,102,106,152–166^ or V.^37,44,51,57,66,67,74,75,82,98,106,138,148,164,165,167–170^ Though not always significantly, lung function in irradiated regions was usually found to worsen,^37,51,57,59,66,72,82,98,102,153–161,163,164,167,169–171^ except in regions to which function was shunted^72,102,154,155,169^ or where tumor shrinkage restored air or blood flow.^74,75,82,138,148,156,157,161,164,165,170^

These results were not, however, always tied to other metrics of lung function. Abratt and Willcox^158,159^ did not find correlations between changes in perfusion and changes in pulmonary function test (PFT) results; however, several other studies did.^168,169,171^ Marks et al^172^ specifically found that the odds of PFT improvement after RT increases only if the tumor is central with adjacent hypoperfusion. Others found a correlation between single-acquisition FLI and various PFT results (including forced expiratory volume and diffusing capacity of the lungs for carbon monoxide).^30,35,48,50,58,62,67,100,128,132,136,152,169,173–175^ Multiple studies found correlations between dose-function metrics and changes in PFT scores.^166,176,177^

### Functional lung avoidance: Treatment planning

Fifty-nine studies tested the idea of pretreatment avoidance planning to preferentially spare FL tissue and preserve lung health during RT.^18,21,31,39,53,54,57,64,68,69,71,76,77,80,81,83,85–89,91–95,97,107–120,122,127,129,131,133–137,139,145,146,148–151,162,165,178^ Of these, 48 studies compared pairwise RT plans for real (nonsimulated) patients, 1 of which was optimized using objectives intended to spare standard organs at risk (OARs) and meet standard criteria, and 1 of which used additional objectives pertaining to minimizing damage to FL for the same RT modality and in the same patient.^21,31,39,53,57,64,68,69,71,76,77,81,83,85–87,89,91,92,97,113–120,122,127,131,133–137,139,145,146,148–151^ From these, studies that provided data on dose-function parameters specifically can be seen in Table 1. We did not include results relating changes in dose-function parameters to change in incidence or risk of RILT, as these results are better summarized in the subsequent section (Table 2). Most, but not all, functional plans resulted in a reduction in functional parameters: mean dose to functional lung (fMLD) and percent volume of functional lung receiving ≥ xGy of irradiation (fVx). RT modalities of these studies varied widely and included various dose/fractionation schedules, including stereotactic body RT (SBRT). Three studies reported on SBRT specifically and found that avoidance planning for SBRT similarly results in a reduction in functional parameters, with the exception of fV13.5 for Vicente et al.^57,116,120^ Some studies had a mix of schedules in their cohorts and did not break down results by technique/fractionation. As such, we did not have enough information to characterize the effect of this on our results.

Studies in Table 1 that also reported on dose to OARs had varying results. In many cases, but not all,^21,39,57,64,69,71,81,83,85–87,89,91,107,109,110,113,115,116,118,119,131,133–137,145,148,149,151^ improvement to functional parameters came at the cost of decreased target volume coverage and/or increased OAR doses.^21,39,71,81,86–89,92,109–112,114,116–120,133,136,139,145,146,149^ In these cases, OARs that sustained increased doses were lung,^21,117,145,149^ esophagus,^21,71,87,114,145,146^ heart,^21,71,89,133,146^ and spinal cord.^21,81,89,92,111,118,119,139,145,146^

Sixteen studies directly compared different methods of RT delivery, within the scope of FL avoidance planning.^21,57,69,83,86,87,92–95,109,117,122,127,131^ Of these, studies that provided data on dose-function parameters specifically can be seen in Table 3. Proton beam therapies (PBT) consistently outperformed photon-based therapies in terms of functional parameters.^86,92,93^ Dougherty et al^86^ and Huang et al^92^ found this was accompanied by an improvement in PTV and OAR parameters, whereas Ieko et al^93^ found this came at a small but significant decrease to PTV D99 (percentage of dose covering 99% of PTV). For photon-based therapies, intensity modulated radiation therapy (IMRT) generally, but not always, outperformed both volumetric modulated arc therapy (VMAT)^57,87,109,128^ and 3-dimensional conformal RT^87,94,95,131^ in functional parameters. This was also generally, but not consistently, accompanied by improvements to PTV and OAR parameters. Finally, several studies compared different beam arrangements for IMRT plans. These results were not consistent. One study found a decrease in only fMLD,^117^ whereas others found a mix of increases and decreases when beam number was reduced.^122,127^ The substantial heterogeneity between studies in terms of RT modality, FLI modality, cancer type, cancer stage, and tumor location can be significant: Vinogradskiy et al^129^ found that dose-function parameters were substantially higher in stage III patients than in stage I using CT V (HU). Kida et al^77^ explored the effect of FLI modality on treatment planning. They found a strong linear correlation between fV20 for CT V (HU) and CT V (Jacobian), each with SPECT V (R = 0.94 and R = 0.85, respectively).^77^ For OAR, PTV, and functional metrics, they found that the differences between FLI modalities were usually under 1%.^77^

Different studies also used different plan optimization methods, which have the potential to influence results. Faught et al^88^ explored knowledge-based planning, which is meant to better spare OARs and meet coverage requirements. They found that compared with conventional, inverse-planned FL RT plans, their method significantly reduced fV20, fMLD, V20 (percent of non-PTV lung volume receiving 20 Gy or more), MLD (mean dose to non-PTV lung), and mean dose to the esophagus. Vicente et al^53^ introduced functionally weighted airway sparing, an optimization algorithm that prioritizes protection of airways in addition to FL tissue on CT scan, and found that it improved V preservation compared with nonairway-sparing functional optimization.^53,57^ Some data were excluded from Table 1 for reporting on functional plans made at different timepoints. Of note, Bucknell et al^82^ explored the use of ^68^Ga-PET (V/Q) to assess FL volumes and adapted treatment accordingly at week 4. This was intended to account for potential changes in functionality that may occur during treatment.^82^ They found a statistically significant decrease in fV20 for ventilation at week 4 for the adaptive plan.^82^ Yamamoto et al^148^ found a similar pattern, with midtreatment functional adaptation further reducing functional dose parameters.

### FL avoidance: Meta-analysis

A total of 22 studies were included in the meta-analysis on mean difference of dose-functional parameters between functional and anatomic plans in the same patient.^39,83,85–87,91,107,109–111,113–116,118–120,122,133,136,137,139^ Dose-functional parameters fV20 and fMLD were chosen, as they were most frequently reported upon and have dose-volume analogs most commonly used in treatment planning. We chose to use the more commonly reported threshold definition of FL, and studies using the weighted method were excluded. Seven studies directly reported either raw data or the required statistics.^83,87,111,122,133,137,139^ Four studies’ authors were able to provide us with data for the analysis.^86,109,118,119^ Three studies reported the required statistics graphically,^85,107,114^ and 5 studies’ means and standard errors (SEs) were derived using *P* values.^91,113,115,120,136^ Three studies reported medians and ranges and were thus kept.^39,110,116^ Nine studies reported multiple different methods or definitions of FL, in which case only 1 outcome was included.^82,86,87,110,111,114–116,133^ Two studies had 1 patient each excluded because of clinically unacceptable plans.^83,139^ A detailed list of exclusions can be found in supplementary table E4.

**Fig. 2.**
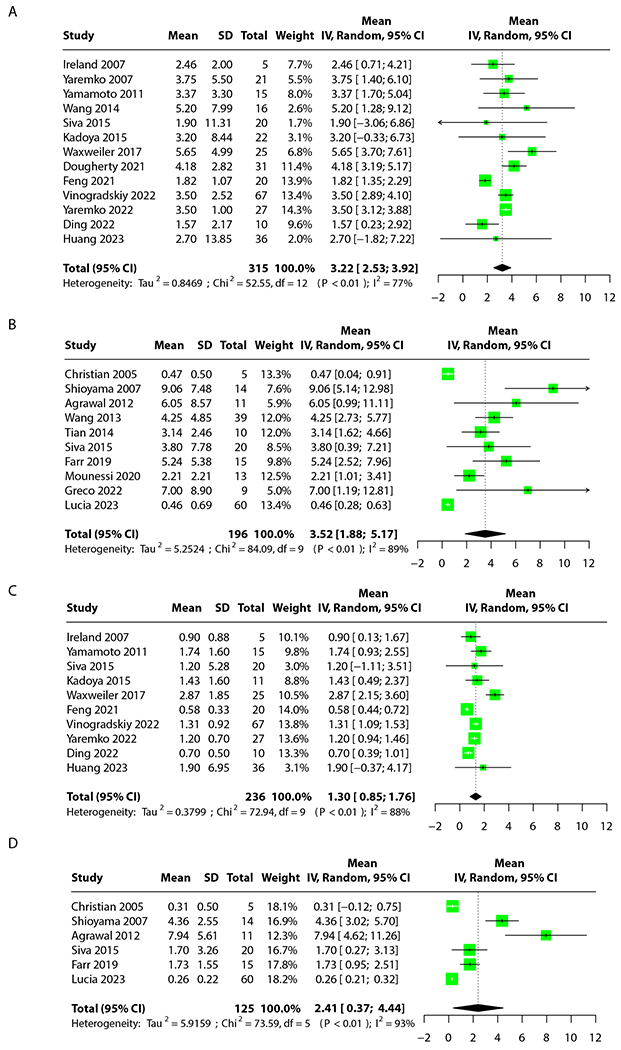
Meta-analysis results. (A) Ventilation dose parameter 20 to functional lung. Twelve studies analyzed across 300 patients. (B) Perfusion dose parameter 20 to functional lung. Ten studies analyzed across 196 patients. (C) Ventilation mean dose to functional lung. Nine studies analyzed across 221 patients. (D) Perfusion mean dose to functional lung. Six studies analyzed across 125 patients.

The results of the meta-analysis can be seen in Figure 2. We report mean differences for fV20 and fMLD, split between perfusion (perf-) and ventilation (vent-)-defined FL. For all 4 parameters, functional avoidance planning resulted in statistically significant reductions: vent-fV20 absolute reduction of 3.22% (95% CI, 2.53-3.92); perf-fV20 absolute reduction of 3.52% (95% CI, 1.88-5.17); vent-fMLD absolute reduction of 1.3 Gy (95% CI, 0.85-1.76); and perf-fMLD absolute reduction of 2.41 (95% CI, 0.37-4.44). Heterogeneity was high, at 77% for vent-fV20, 89% for perf-fV20, 88% for vent-fMLD, and 89% for perf-fMLD. To assess for risk of bias, the JBI analytical cross-sectional study checklist was used.^183^ The average score for each analysis was: 7.37 of 8 (vent-fV20), 6.1 of 8 (perf-fV20), 7 of 8 (vent-fMLD), and 6.3 of 8 (perf-fMLD), indicating low risk of bias.

### FL avoidance definitions: Sensitivity and metaregression

The results for the sensitivity analysis were not statistically significant, indicating that the varying definitions of FL did not influence the results of the meta-analysis. They can be found in Figure E2.

To assess the effect of FL definition on dosimetric parameters directly, instead of on differences between functional and anatomic plans, we then performed metaregression on studies that gave a specific, thresholded definition of FL. A total of 8 studies’ avoidance plans were included: 7 for fV20-vent^71,86,111,115,118,119,131^ and 5 for fMLD-vent.^71,107,111,119,131^ Because of a lack of studies that reported the required statistics for Q, those results were excluded. Several studies provided multiple definitions and parameters. Anatomic plans were not included. The results of the metaregression can be seen in Figure E3. A trend of decreased fV20 or fMLD was seen for higher cutoff thresholds. However, this trend was only statistically significant for fV20-vent (*P* = .0183) and not for fMLD-vent (*P* = .3670). Again, the JBI analytical cross-sectional checklist^183^ was used, with average scores of 7.4 of 8 (vent-fV20) and 7.2 of 8 (vent-fMLD), indicating low risk of bias overall. A detailed list of exclusions can be found in supplementary table E4.

### Prediction of RILT

Thirty-six studies used FL information to generate a predictive model or prediction of risk for developing RILT.^21,50,72–77,84,86,89,90,96,97,99,100,101,103–111,121,123,126,130,133,135,136,140–144,147,174,182,181^ Table 2 details all such studies, which involve a variety of fractionation schedules including SBRT. Results were inconsistent, with some studies finding that the addition of functional parameters enhanced prediction or reduced incidence of RILT-related outcomes,^50,84,86,90,96,97,99,103,105,121,123,130,135,136,140–142,144,147^ whereas others did not.^21,79,84,100,104,106,126,133,141,182,181^

To better quantify the relationship between grade 2+ RP and FL irradiation, we analyzed how incorporating functional information can enhance grade 2+ RP prediction. A total of 10 studies were included: 7 for anatomic-only models,^79,84,89,105,121,130,144^ 6 for models including Q information,^79,84,99,121,130,144^ and 5 for models including V information.^89,101,105,143,144^ Multiple studies included models across multiple categories. A detailed list of exclusions can be found in supplementary table E4.

**Fig. 3.**
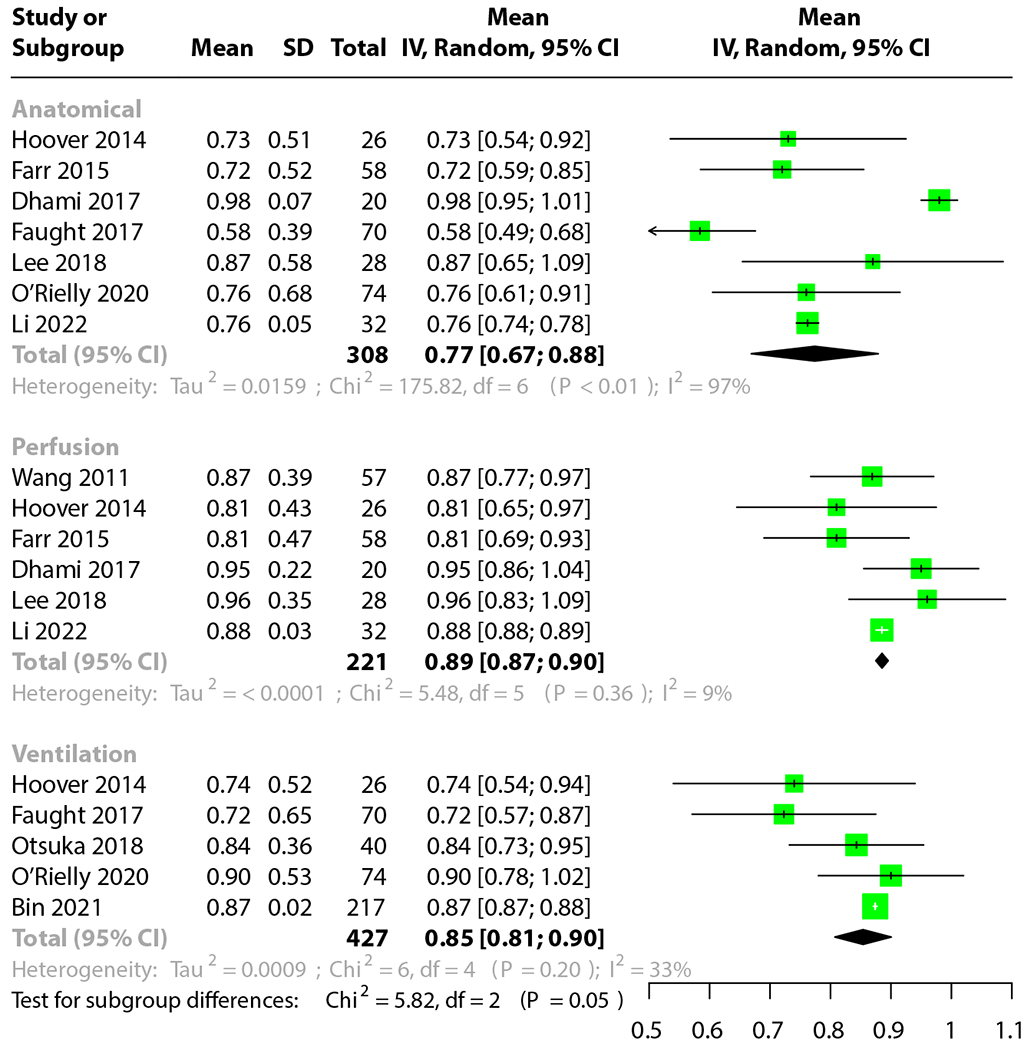
Meta-analysis of the area under the curve for prediction of grade 2+ radiation pneumonitis. Anatomic, perfusion, and ventilation-based models presented.

The results of the analysis can be seen in Figure 3. The anatomic models had a mean AUC of 0.77 (95% CI, 0.67-0.88). The Q-including models had a mean AUC of 0.89 (95% CI, 0.87-0.90). The V-including models had a mean AUC of 0.85 (95% CI, 0.81-0.90). These results were not statistically significant because of the CI overlap. Notably, the anatomic models had a high χ^2^ value, indicating substantial interstudy variability. This was not seen with the Q and V models. The case series JBI checklist tool^184^ was used, with an average score of 7.7 of 10.

### Interventional clinical trials

Recently, several interventional, multipatient clinical trials that employ functional avoidance planning have been published. The results of these 9 studies can be seen in Table 4.^76,82,102,119,124,126,133,136,148^ Only 2 studies included an anatomic arm to compare to the functional planning cohort.^102,133^ Of these, results were inconsistent: Thomas et al^102^ found a statistically significant reduction in dose-function parameters for plans used for treatment, but Yaremko et al^133^ did not. However, neither study reported any significant worsening of OAR or PTV metrics in the functional cohort compared with the anatomic cohorts. All single-arm studies that compared functional plans to anatomic plans computed for reference found reductions in dose-function parameters.^76,82,102,119,133,136,148^ Importantly, Miller et al^124^ found not only a reduction in functional parameters (as per a primary analysis by Vinogradskiy et al^119^), but also that the avoidance plans resulted in significantly better postlung RT PFT scores than the historical control. Similar to the results of the 2-arm comparative trials, the OAR and PTV metrics for functional plans were not significantly worse than anatomic plans, aside from a 1.4 Gy-increase in the cord max dose in Vinogradskiy et al.^119^ Two single-arm studies that assessed rates of RP found that functional planning significantly reduced the rate of grade 2+ RP^119,136^ relative to the historical control. Yaremko et al^133^—1 of the 2-arm studies—found no significant difference in grade 2+ RP between the 2 cohorts, though this result should be interpreted with caution, as the study was closed before full accrual and was not powered to detect RP differences.^133^

**Fig. 4.**
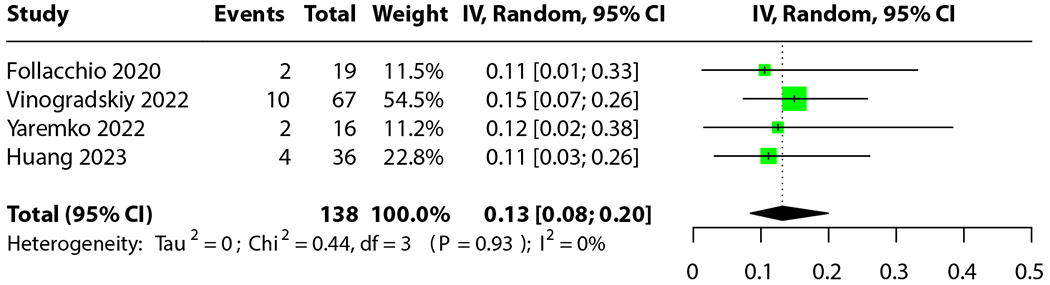
Meta-analysis of incidence of grade 2+ radiation pneumonitis for intensity modulated radiation therapy and volumetric modulated arc therapy treated cohorts with functional avoidance planning.

Five interventional studies reported the rate of grade 2+ RP for the avoidance cohort.^76,119,126,133,136^ A detailed list of exclusions can be found in suppplementary document E4. Notably, Thomas et al^126^ used PBT on a substantial proportion of their cohort. We deemed this a confounding variable on an already small data set, as the remaining studies used only IMRT and VMAT. Excluding Thomas et al, we conducted a meta-analysis on the rate of grade 2+ RP for the remaining 4 studies. The results can be seen in Figure 4: for all interventional trials to-date, the incidence of grade 2+ RP for patients treated with IMRT or VMAT is 13% (95% CI, 8%-20%). The range was 11% to 15%, and the median was 11.5%. For all studies except Yaremko et al,^133^ we used the JBI quasi-experimental checklist.^185^ The mean score was 7.4 of 9. For Yaremko et al, we used the JBI randomized controlled trial checklist,^184^ for which the study achieved a perfect score.

## Discussion

This review provides a comprehensive update to the state of knowledge relating to FLI for RT planning in lung cancer. We employed a variety of statistical methods to substantially increase the size of the previous meta-analysis and include an in-depth analysis of the significance of FL definition to study results. In addition, we provide a new analysis on the relationship between FLI and grade 2+ RP.

The 2 main purposes of this review were to investigate the difference in functional parameters between anatomic and functional plans and the ability of functional parameters to predict grade 2+ RP. The results of our analyses further solidify that avoidance planning substantially improves sparing of functional lung compared with anatomic plans for the same patient. This is the cornerstone of the integration of FLI into RT planning: all posited benefits of functional avoidance planning hinge on the concept that such planning will preferentially spare critical regions.

Our second outcome was less definitive. A previous meta-analysis on rates of grade 2+ RP for patients treated with standard chemoradiation prior to the use of adjuvant immune checkpoint inhibitors reported a rate of 29.8%.^186^ Our meta-analysis indicates that photon-based avoidance planning results in a significantly lower rate of 13% (95% CI, 8%-20%). Although some of the studies included patients treated with durvalumab, no durvalumab-era meta-analyses for grade 2+ RP rates exist for comparison. However, this drug typically is found to increase rates of grade 2+ RP,^187,188^ indicating that our historical control may be a conservative estimate and that our results likely maintain significance, though this should be balanced with an understanding that RT delivery techniques have also improved since then. Although the use of avoidance planning statistically significantly lowered functional parameters (Fig. 2) and thus decreased the rate of grade 2+ RP, we failed to find a statistically significant trend of increased predictive power of grade 2+ RP for models that incorporate FL information. The existence of a trend suggests that an increase in sample size may help determine whether there is a true difference between these groups of predictive models or not. Importantly, these 2 results are not in direct contradiction, but indicate that the full nature of the relationship between FL irradiation and grade 2+ RP may not yet be fully elucidated.

### Recommendations: FLI modalities

The wide variety of FLI modalities available highlights a recurring issue: there is no agreement on the optimal modality for incorporation into RT planning. SPECT, PET, DECT, and MRI are expensive but are established methods of acquiring lung function measures, particularly DECT-Q as validated against SPECT-Q, with strong correlations.^7,11^ MRI and PET were not compared directly to the SPECT standard in any study.

CT-based methods, on the other hand, present cost-effective and convenient ways of extracting functional information from a scan that is already standardly required in the management of patients with lung cancer. However, as discussed, CT-V is inconsistent in correlation with SPECT,^10,13,24–26,28–30,36^ and no thoroughly validated Q analog currently exists. The previous review on CT-V corroborates this inconsistency.^70^

The substantial heterogeneity between all these methods, and even between V and Q SPECT,^9,12,22^ highlights the importance of homing in on 1 standard-of-care method to avoid confounding results. We thus recommend that the development of CT-V continues. The results of a recent clinical trial demonstrating a reduction in grade 2+ RP compared with historical control using CT-V^119^ suggests that although inconsistent, this method does show potential as a viable, convenient, and inexpensive alternative to other methods. We further recommend that future interventional trials consider using CT-V as one method of FL imaging, to allow for a more in-depth examination of the utility of this method.

### Recommendations: Definitions

As with FLI modalities, definitions of FL vary widely between papers, raising concerns around synthesizing information between studies to determine the potential of FLI. The sparsity of investigation into the best definition is perhaps one of the largest gaps in the FLI literature at this point. We attempt to investigate this through our sensitivity and metaregression. Our sensitivity analysis shows that the definition of FL, for relevant studies, was not significant to the results of our meta-analysis. In contrast, our metaregression showed that in functional avoidance plans, when the cutoff value for defining lung as functional is low, functional parameters trend higher—though this trend only reached significance for fV20-vent. In some cases, this trend could be caused by the small volume of FL in the high-cutoff group, which may be easier to avoid. However, this may depend on FL location relative to the tumor: if they are too close, FL sparing will be challenging. Although the observed relationship in the metaregression may be cohort-dependent, it suggests that functional plans with higher cutoff values may see greater sparing of FL. In combination with the sensitivity analysis, it indicates that although the difference between functional and anatomic plans may not change based on FL definition, the raw value of the functional parameters may change. Further investigation into this trend, and for both V and Q, is thus warranted to determine whether it is true or spurious. Such investigation should also consider the viability of other definitions, such as the less commonly used weighting method, which has demonstrated predictive ability for RILT as well.^50,73,78,90,97,99,104,121,123,126,140–144,147^ We thus recommend that future studies investigate multiple different definitions of FL to contribute to a currently sparse pool of data.

### Recommendations: Toxicity reduction

Information regarding the incidence of RILT in relation to FL irradiation, as well as the prediction of RILT using FL information, is suggestive but inconsistent. Current clinical trials indicate that avoidance planning reduces the rate of grade 2+ RP, as corroborated by our meta-analysis for VMAT and IMRT. However, when it comes to prediction, some studies show improved predictive ability compared with anatomic parameters, ^50,84,86,90,96,97,99,103,105,121,123,130,135,136,140–142,144,147^ although others do not.^21,79,84,100,104,106,126,133,141,182,181^ This is further corroborated by the inconclusive results of our own analysis, which may be improved with a larger data set.

We thus recommend that investigation of the relationship between avoidance planning and RILT continue. However, full reporting of all parameters and error bars related to results is critical for proper synthesis of information. We implore authors to report full details of models, including cutoff values for normal tissue complication probability models, 95% CIs, and the appropriate descriptive statistics for odds ratios, to facilitate future collaborative analyses.

Additionally, some—though not all—planning studies found that that FLI has the potential to raise doses to the lung,^21,117,145,149^ esophagus,^21,71,87,114,145,146^ heart,^21,71,89,133,146^ and spinal cord.^21,81,89,92,111,118,119,139,145,146^ Even if FLI reduces the rate of RILT, its benefit may be mitigated by an increase in other organ toxicities. This tradeoff is critically important and must be elucidated to determine the overall benefit of FLI for RT treatment planning. We thus further suggest that authors also report nonlung OAR dosimetry and toxicity outcome differences between conventional and avoidance techniques.

### Recommendations: Future trials

There is increasing interest in prospective trials integrating FL avoidance planning. Most trials to date are single arm, and these largely found improvements compared with historical means and anatomic plans.^82^ However, 1 contemporary trial that incorporated PBT had a markedly high rate of grade 2+ RP,^126^ while another study found an improvement in functional parameters compared with anatomic plans but found no substantial benefit to midtreatment functional adaptation using those plans.^82^ The only 2-arm trials provide conflicting results, with one finding improvements between cohorts^102^ and the other finding no significant difference.^133^

Prospective interventional trials represent the best opportunity to fully understand the effect of avoidance planning on the development of RILT. Although our meta-analysis indicates that IMRT and VMAT avoidance planning may significantly reduce the rate of grade 2+ RP, comparison to older review articles and historical controls is not ideal, as this does not control for advances in technologies, administration of treatments, and differences between cohorts. We thus recommend the use of comparative trials, particularly randomized study designs, when possible, to better understand the effect of FL avoidance on RILT and other clinical outcomes. Current clinical trials registered on clinicaltrials.gov include 1 2-arm trial (National Clinical Trial [NCT]05302817^189^) and 3 1-arm trials (NCT05134558,^190^ NCT02773238,^191^ NCT02492867^192^), 2 of which aim to assess the incidence of RILT as a result of the intervention.

### Limitations: Literature

Although the included studies report on a wealth of knowledge regarding FLI in the context of lung cancer, the individual studies do have some limitations. Apart from established concerns about small sample size, it is important to keep in mind that nearly every planning study provided few details on the rationale for its chosen FL definition. With little existing data on the most robust and RILT-predictive definition, the utility of individual study results must be taken with caution.

Furthermore, of the predictive modeling studies provided, almost none conducted external validation of their models. Although the predictive performance of FL information is important to assess, trained and fitted models that have not been tested on validation data should not be considered wholly complete. As such, there is an inherent bias to presenting a model that has only been tested on data from the cohort it was originally fitted to.

Finally, although the studies included in the tables do come from a variety of continents, medical centers, and populations, those that responded to our requests for data were exclusively authors at Western institutions. As a result, many studies from non-Western institutions, particularly Asia, had to be excluded from our analyses, introducing an inherent under-representation of particular populations of patients with lung cancer and health care systems in the statistical analyses.

Overall, by completing the relevant JBI checklists, we found that studies generally had low risk of bias. However, we acknowledge that this is not a foolproof or complete method, and that—as always—results should be taken in-context and with caution.

### Limitations: Review design and statistical analysis

Our review design is not without limitations. To begin, some of our analyses have small sample sizes, and for others, approximations had to be made for them to be included. Although we attempt to account for the small sample sizes using the appropriate weighting techniques, it remains that the results of these studies may be skewed because of the small number of patients involved. Furthermore, the studies included in this review have heterogeneous lung cancer types, RT modalities, FLI modalities, and FL definitions. We attempted to mitigate this where possible, by eliminating studies that used the weighting FL definition, creating a strict cutoff for the metaregression to account for different threshold-determining methods, and by removing proton therapy for the interventional FLI trial meta-analysis of RP rates. Although all these decisions improve the uniformity of the data, some degree of heterogeneity remains, as there are insufficient papers to independently analyze and control for every possible combination of the confounders. Our study is also limited by the inconsistent choice of reported statistics: although our tables are large, the number of studies in each table that make it to the analysis is small, because many studies do not report the required statistics for the analysis—a byproduct of our attempt to control for the aforementioned variables of concern. Conclusions drawn should thus be interpreted with caution. Finally, all of our functional parameter meta-analyses had high heterogeneity, again indicating that results should be interpreted with caution.

## Conclusions

FLI demonstrates substantial potential to spare functioning lung in patients with lung cancer. However, the costs of additional pretreatment scans and potential technical challenges^70^ necessitate an in-depth examination of any potential benefit that incorporation of FLI into RT may have for lung cancer. Our understanding of this field is currently limited by inconsistency in definition of FL, FLI modality used, and reporting of relevant post-RT outcomes. An increased number of interventional trials, with more on the way, will provide for a more direct approach to understand the effect of avoidance planning on dosimetric parameters and clinical outcomes.

## Supplementary Material

Sup3

Sup2

Sup1

Sup4

Sup5

Sup6

Sup7

Sup8

## Figures and Tables

**Table 1:** Comparison of functional parameters, PTV, and OARs for pairwise anatomic and functional-avoidance RT plans

Study first author	N	Age	Cancer type	FLI modality: Definition	RT modality	Dose-function parameters: Absolute reduction (anatomic - functional, mean difference)	Plan quality: Significant results (anatomic - functional)
Agrawal^137^	11	N/A	All NSCLC	SPECT Q: Visual thresholding into 2 groups	3DCRT	fV20 ↓ 5.45 fV30 ↓ 7.54* fMLD ↓ 7.72*	V20 ↓ 5.86* V20 ↓ 7.59* MLD ↓ 7.76*
Christian^83^	6	N/A	All NSCLC	SPECT Q: Visual thresholding into 2 groups; weighting	3DCRT	fV20 ↓ 0.807 (coplanar) fV20 ↓ 0.597 (noncoplanar) fMLD ↓ 0.325 (coplanar) fMLD ↑ 0.378 (noncoplanar) Mean perfusion-weighted lung dose (MPWLD): ↓ 0.03 (coplanar) MPWLD: ↑ 0.118 (noncoplanar)	No significant difference
Ding^85^	10	58.5 (med)	All NSCLC	Xe-MRI V: Thresholded into 4 groups (unspecified)	IMRT	fV5 ↓ 3.5* fV10 ↓ 2.7* fV20 ↓ 1.5* fMLD ↓ 0.7	No significant difference
Doi^151^	12	66 (med)	Malignant pleural mesothelioma (MPM)	CT V: Direct thresholding at −860 HU	VMAT	Medians reported fV5 ↓ 9.8* fV10 ↓ 4.9* fV20 ↓ (difference not reported) fMLD ↓ 0.6*	Medians reported V5 ↓ 9.8* MLD ↓0.6*
Dougherty^86^	31	64 (med)	26 NSCLC 5 SCLC	CT V (HU): 15% of max, with further processing	IMPT VMAT	fV5 ↓ 2.1* (VMAT) fV5 ↓ 1.52* (IMPT) fV10 ↓ 8.88* (VMAT) fV10 ↓ 4.27* (IMPT) fV20 ↓ 3.72* (VMAT) fV20 ↓ 4.11* (IMPT) fV30 ↓ 2.01* (VMAT) fV30 ↓ 2.6* (IMPT) fV40 ↓ 1.26* (VMAT) fV40 ↓ 1.69* (IMPT) fV50 ↓ 0.51 (VMAT) fV50 ↓ 0.96* (IMPT) fMLD[RBE] ↓ 1.56* (VMAT) fMLD[RBE] ↓ 1.51* (IMPT)	CTV max ↑ 0.77* (IMPT) CI ↓ 0.07* (VMAT) CI ↓ 0.08* (IMPT) HI ↑ 0.01* (VMAT) HI ↑ 0.01* (IMPT) MLD ↓ 0.86* (VMAT) MLD ↓ 0.95* (IMPT) V20 ↓ 2.07* (VMAT) V20 ↓ 2.48* (IMPT) V5 ↓ 1.91* (VMAT) V5 ↓ 2.13* (IMPT) Mean esophagus ↓ 0.69* (VMAT)
Farr^87^	15	71 (med)	12 NSCLC 1 SCLC 2 other	SPECT Q: 20%, 40%, 60%, and 80% of max	IMRT VMAT 3DCRT	20% threshold fV5 ↑ 0.41 (IMRT) fV5 ↓ 0.85 (VMAT) fV5 ↑ 1.86 (3DCRT) fV20 ↓ 2.69* (IMRT) fV20 ↓ 1.89* (VMAT) fV20 ↓ 0.14 (3DCRT) fV30 ↓ 1.35* (IMRT) fV30 ↓ 0.24 (VMAT) fV30 ↓ 1.09 (3DCRT) fMLD ↓ 0.75* (IMRT) fMLD ↓ 0.43 (VMAT) fMLD ↑ 0.26 (3DCRT) 40% threshold fV5 ↓ 1.74 (IMRT) fV5 ↓ 2.05 (VMAT) fV5 ↑ 1.78 (3DCRT) fV20 ↓ 5.24* (IMRT) fV20 ↓ 4.52* (VMAT) fV20 ↓ 2.73* (3DCRT) fV30 ↓ 3.03* (IMRT) fV30 ↓ 1.67 (VMAT) fV30 ↓ 3.25 (3DCRT) fMLD ↓ 1.73* (IMRT) fMLD ↓ 1.18* (VMAT) fMLD ↓ 0.37 (3DCRT) 60% threshold fV5 ↓ 6.5* (IMRT) fV5 ↓ 3.15 (VMAT) fV5 ↑ 2.27 (3DCRT) fV20 ↓ 4.95* (IMRT) fV20 ↓ 5.57* (VMAT) fV20 ↓ 2.21 (3DCRT) fV30 ↓ 3.36 (IMRT) fV30 ↓ 1.85 (VMAT) fV30 ↓ 2.17 (3DCRT) fMLD ↓ 2.15* (IMRT) fMLD ↓ 1.57* (VMAT) fMLD ↓ 0.13 (3DCRT) 80% threshold fV5 ↓ 9.5* (IMRT) fV5 ↓ 2.92 (VMAT) fV5 ↑ 0.31 (3DCRT) fV20 ↓ 3.36* (IMRT) fV20 ↓ 4.64 (VMAT) fV20 ↓ 2.78 (3DCRT) fV30 ↓ 1.86 (IMRT) fV30 ↓ 3.62 (VMAT) fV30 ↓ 2.53 (3DCRT) fMLD ↓ 2.3* (IMRT) fMLD ↓ 1.73* (VMAT) fMLD ↓ 0.52 (3DCRT)	V20 ↓ 2.02* (IMRT) V30 ↓ 1.03* (IMRT) Esophagus V60 ↓ 1.5* (IMRT) Esophagus V60 ↑ 1.29* (VMAT)
Faught^89^	70	N/A	All NSCLC	CT V (HU): 15% below average lung function	IMRT	Significance not reported fV5 ↓ 4 fV10 ↓ 6.2 fV20 ↓ 3.3 fV30 ↓ 4.3 fMLD ↓ 1.2	Significance not reported Cord max ↑ 3.43 V20 ↓ 0.98 MLD ↓ 0.08 Mean esophagus ↓ 0.17 Heart V60 ↑ 0.11 Heart V45 ↑ 1.63 Heart V40 ↑ 3.18
Feng^39^	20	64 (med)	All lung cancer	CT V (Jacobian with density): Jacobian thresholded at 60% and 30%-60% of max; density thresholded at >−700 HU and −850 to 701 HU	IMRT	Jacobian fV5 ↓ 2.07* fV20 ↓ 1.82* fV30 ↓ 0.88* fVMLD ↓ 0.59* Density fV5 ↓ 2.16* fV20 ↓ 2.16* fV30 ↓ 1.1* fMLD ↓ 0.57*	V5 ↓ 1.48* V20 ↓ 2.26* V30 ↓ 0.48* MLD ↓ 0.37* PTV mean ↑ 0.59* PTV D_2_ ↑ 1.07* PTV D_98_ ↓ 0.54* CI ↓ 0.06* HI ↑ 0.02*
Greco^91^	9	72 (med)	All lung cancer	SPECT Q: Thresholded at 70% and 40%-70% of max	3DCRT	fV20 ↓ 7* ipsi-fV20 ↓ 16* ipsi-fMLD ↓ 4*	No significant difference
Hodge^135^	1	N/A	NSCLC	He-MRI V: Automated threshold	N/A	Significance not reported. Mean Normalized total dose (NTD) to functional lung ↓ 4.01	Significance not reported. Mean NTD to total lung↓ 1.45
Huang^92^	8	66 (med)	NSCLC	CT V (Jacobian): Thresholded into 2 groups (unspecified)	Double scattering proton therapy (DSPT) IMPT	Significance not reported. fV5 ↓ (DSPT, IMRT) fV20 ↓ (DSPT, IMRT) fMLD ↓ (DSPT, IMRT)	Significance not reported. Potentially substantial increase seen in cord max dose for functional IMPT plan
Huang^71^	11	58 (mean)	5 NSCLC 1 SCLC 3 esophageal 2 thymoma	CT V (breath change-based): Thresholded at top 20%, 30%, and 40% of max	IMRT	Small tumors fV5 ↓* fV20 ↓* fMLD ↓* Large tumors fV5 ↓* fV20 ↓ fMLD ↓	Significance not reported V5 ↓ 5.8 V20 ↓ 2.32 MLD ↓ 1.05 Heart V40 ↑ 5.12 Mean heart ↑ 0.61 Cord max ↑ 1.73 Esophagus V35 ↑ 3.79 Mean esophagus ↑ 0.88
Huang^136^	36	66 (med)	All NSCLC	CT V (Xe-enhanced): >15 HU	VMAT IMRT	fV5 ↓ 2.1 fV20 ↓ 1.7* fMLD ↓ 2.9*	Maximum dose ↑ 1.5* V20 ↓ 2* MLD ↓ 1.1*
Ireland^139^	6	N/A	All NSCLC	He-MRI V: Manual thresholding	IMRT	fV20 ↓ 2.46* fMLD ↓ 0.9*	Cord max ↑ 3.02*
Iqbal^81^	29	N/A	All lung cancer	CT V (density change): Weighted or thresholded at 33% and 66%	IMRT	Percent volume reduction reported. Thresholded method fV20 ↓ 2% fV30 ↓ 7%* fV40 ↓ 8.8%* fMLD ↓ 1.1 Weighted, voxel-wise method fV20 ↓ 7.8* fV30 ↓ 13.5* fV40 ↓ 17.2* fMLD ↓ 6.2*	Percent volume reduction reported. Thresholded method V30 ↓ 5.3* V40 ↓ 7* Weighted, voxel-wise method V20 ↓ 7.1* V30 ↓ 13* V40 ↓ 16.8* MLD ↓ 6.4* Cord max ↑*
Kadoy^120^	11	80 (med)	Peripheral lung tumors	CT V (Jacobian): 90th percentile	3DCRT (SBRT)	fV5 ↓ 8.2* fV10 ↓ 7.3* fV20 ↓ 3.2 fMLD ↓ 1.43*	HI ↑ 0.03*
Kida^77,†^	8	64 (med)	1 SCLC 4 NSCLC 3 Other	CT V (HU) CT V (Jacobian) SPECT V All: weighted	IMRT	Significance not reported HU fMLD ↓ 0.36 fV20 ↓ 1.88 Jacobian fMLD ↓ 0.38 fV20 ↓ 2.03 SPECT fMLD ↓ 0.42 fV20 ↓ 2.17	Not reported
Kimura^69^	8	72.5 (med)	All lung cancer	CT (direct thresholding): > −860 HU	IMRT VMAT	fV30 ↓ 1.3* (IMRT) fMLD ↓ 0.3 (IMRT) fMLD ↓ 0.5 (VMAT)	PTV V5 ↑* (IMRT, VMAT, PTV > 250 cc) PTV fV20 ↑* (VMAT, PTV > 250 cc)
Lee^21^	8	N/A	All NSCLC	SPECT Q: Threshold into 7 equidistant bins; 70% of max threshold used for dose-function parameters	VMAT	Pairwise significance not reported Medians reported; difference between medians used below, fMLD ↓ 7.6 (median)	Pairwise significance not reported. PTV V60 ↑ (median) BTV mean ↑ (median) SUV_peak_ ↑ (median) MLD ↑ (median) V5 ↓ (median) V10 ↓ (median) Heart mean ↑ (median) Heart mean ↓ (median) Heart V40 ↑ (median) Heart V45 ↑ (median) Heart V60 ↑ (median) Cord D_0.03_ ↑ (median) Mean esophagus ↑ (median)
Lucia^116^	60	69 (med)	25 NSCLC 23 secondary 12 other	Ga-PET Q: Minimum volume containing 50%, 70%, and 90% of activity	VMAT (SBRT)	Medians reported. 50% threshold fMLD ↓ 0.2* fV5 ↓ 1.4* fV10 ↓ 0.8* fV15 ↓ 0.5* fV20 ↓ 0.3* 70% threshold fMLD ↓ 0.2* fV5 ↓ 1.3* fV10 ↓ 0.7* fV15 ↓ 0.4* fV20 ↓ 0.2* 90% threshold fMLD ↓ 0.2* fV5 ↓ 1* fV10 ↓ 0.6* fV15 ↓ 0.4* fV20 ↓ 0.1*	MLD ↓ 0.1* V5 ↓ 0.7* V10 ↓ 0.5* V15 ↓ 0.3* V20 ↓ 0.1* PTV coverage ↓*
Matrosic^64^	18	N/A	Lung Mediastinal	CT Parametric response mapping (PRM): Tissue-based contours	VMAT	Emphysema fV20 ↓ 8.95*Small airway disease (SAD) fV20 ↓ 4.9*Parenchymal disease (PD) fV20 ↓ 3.42*	MLD ↓ 0.31* V20 ↓ 1.85*
Matuszak^146^	15	N/A	NSCLC	SPECT Q: Weighted	IMRT	fMLD ↓ 2.7*	Cord functional mean ↑ 5.4* Esophagus functional mean ↑ 3* Heart functional mean ↑ 2.3* Conformity ↓ 17.6%*
McGuire^108^	5	N/A	Lung cancer	SPECT Q: Threshold into 4 regions, with subsequent weighting	IMRT	fV20 ↓ 13.6 (% reduction)* fV30 ↓ 10.5 (% reduction)*	Not reported
McGuire^127^	5	N/A	Lung cancer	SPECT Q: Threshold into 4 regions, with subsequent weighting	IMRT: 7 beams	Significance not reported fV20 ↓ 5.9 fV30 ↓ 2.66	Not reported
Miften^34^	1	N/A	NSCLC	SPECT Q: Manual annotation	IMRT	Significance not reported Functional equivalent uniform dose (EUD) to lungs ↓ 4.21	Significance not reported EUD to lung ↓ 4.23 EUD to heart ↓ 3.45
Mounessi^114^	13	N/A	NSCLC	SPECT Q: 30% of max	IMRT VMAT	All patients fMLD ↓ 0.59 (IMRT) fMLD ↓ 0.16 (VMAT) fV5 ↓ 0.45 (IMRT) fV5 ↓ 3.22 (VMAT) fV10 ↓ 1.25 (IMRT) fV10 ↓ 0.29 (VMAT) fV20 ↓ 3.14* (IMRT) fV20 ↓ 2.02* (VMAT) fV30 ↓ 1.11 (IMRT) fV30 ↓ 0.4 (VMAT) Localized hypo-perfusion fMLD ↓ 0.31 (IMRT) fMLD ↓ 0.12 (VMAT) fV20 ↓ 5.29* (IMRT) fV20 ↓ 2.7* (VMAT) fV30 ↓ 0.37* (IMRT) fV30 ↓ 1.18* (VMAT) Diffuse hypo-perfusion fMLD ↓ 0.75 (IMRT) fMLD ↓ 0.17 (VMAT) fV20 ↓ 1.8* (IMRT) fV20 ↓ 1.62* (VMAT) fV30 ↓ 1.58 (IMRT) fV30 ↓1.18* (VMAT)	Esophagus parameters ↑* (VMAT, IMRT)
Munawar^117^	10	N/A	NSCLC	SPECT V: Threshold at 50% and 70% of max; weighting	IMRT	All patients fMLD ↓ 5.552* Group 1: Clinically acceptable fMLD ↓ 2.26 Group 2: Not acceptable fMLD ↓ 8.844*	All patients MLD ↑ 45.051* Group 1: Clinically acceptable MLD ↑ 9.326 Group 2: Not acceptable MLD ↑ 82.642*
Seppenw-oolde^149^	116	N/A	NSCLC	SPECT Q: Weighted	N//A	Significance not reported. Tumor hypoperfusion fMLD ↓ 4 (fMLD-optimized) fMLD ↓ 2 (fV20-optimized) Tumor-adjacent hypoperfusion fMLD ↓ 4.5 (fMLD-optimized) fMLD ↓ 3 (fV20-optimized) Tumor-ventral hypoperfusion fMLD unchanged (fMLD-optimized) fMLD unchanged (fV20-optimized) Ipsilateral lung hypoperfusion fMLD ↓ 2 (fMLD-optimized) fMLD ↑ 0.5 (fV20-optimized) Misc. fMLD ↓ 10 (fMLD-optimized) fMLD ↓ 10 (fMLD-optimized)	Significance not reported. Tumor hypoperfusion MLD ↓ 7.5 (fMLD-optimized) MLD ↓ 3 (fV20-optimized) V20 ↑ 1 (fMLD-optimized) V20 ↓ 2.5 (fV20-optimized) Tumor-adjacent hypoperfusion MLD ↓ 1.5 (fMLD-optimized) MLD ↓ 0.5 (fV20-optimized) V20 ↓ 3.5 (fMLD-optimized) V20 ↓ 3.5 (fV20-optimized) Tumor-ventral hypo-perfusion MLD 0.5 (fMLD-optimized) MLD unchanged (fV20-optimized) V20 unchanged (fMLD-optimized) V20 unchanged (fV20-optimized) Ipsilateral lung hypo-perfusion MLD ↓ 1.5 (fMLD-optimized) MLD ↑ 1 (fV20-optimized) V20 ↓ 2.5 (fMLD-optimized) V20 ↓ 3 (fV20-optimized) Misc. MLD ↓ 1.5 (fMLD-optimized) MLD ↓ 1.5 (fMLD-optimized) V20 ↓ 17 (fMLD-optimized) V20 ↓ 17 (fV20-optimized)
Shioyama^110^	14	62 (med)	15 NSCLC 1 neuroendo	SPECT Q: 50th and 90th percentiles	IMRT	Median differences reported. 50% threshold fMLD ↓ 2.2* fV5 ↓ 7.1* fV10 ↓ 6* fV20 ↓ 5.1* 90% threshold fMLD ↓ 4.2* fV5 ↓ 7.1* fV10 ↓ 12* fV20 ↓ 6.8*	Median differences reported. CI ↑ 0.2* PTV min ↓ 0.1*
Siva^111^	20	68 (med)	NSCLC	Ga-PET V/Q: Perfusion 70th percentile; ventilation 70th and 50th percentile	IMRT	Well perfused fMLD ↓ 1.7* fV5 ↓ 13.2* fV10 ↓ 7.3* fV20 ↓ 3.8* fV30 unchanged fV40 ↓ 0.63 fV50 ↓ 0.3 fV60 ↑ 0.05 Well-ventilated fMLD ↓ 1.2 fV5 ↓ 9.10 fV10 ↓ 5.3 fV20 ↓ 1.9 fV30 ↓ 0.8 fV40 ↓ 0.19 fV50 ↑ 0.14 fV60 ↓ 0.08 Ventilated fMLD ↓ 0.4 fV5 ↓ 5.2 fV10 ↓ 2.5 fV20 ↓ 0.3 fV30 ↓ 0.2 fV40 ↑ 0.09 fV50 ↑ 0.09 fV60 ↓ 0.12	Cord max ↑* for high-perfusion plan
Siva^112^	14	N/A	NSCLC	Ga-PET Q: top 70% of voxels	3DCRT	Median differences reported Perfused lung fV5 ↑ 3.33* fV20 ↑ 0.42 fV30 ↓ 1.07 Hypo fV40 ↓ 1.15 fV50 ↓ 1.27 fV60 ↓ 1* fMLD ↓ 0.07 Well-perfused lung fV5 ↑ 0.15 fV20 ↓ 0.46 fV30 ↓ 1.76* fV40 ↓ 1.25* fV50 ↓ 0.74* fV60 ↓ 0.99* fMLD ↓ 0.86*	Not reported
St-Hilaire^150,†^	15	N/A	All lung cancer	SPECT Q: Weighting	IMRT	Local defects fV20 ↓ 1.13 fMLD ↓ 0.9 Nonuniform fV20 ↓ 1.69 fMLD ↓ 0.69* All patients fV20 ↓ 1.43 fMLD ↓ 0.79*	Not reported
Tian^122^	10	N/A	NSCLC	SPECT Q (7-beam): Threshold (unspecified)	IMRT	Significance not reported fV20 ↓ 3.14 fV30 ↓ 0.7	Not reported
Vicente^57^	12	64	All lung cancer	CT V (HU-Jacobian hybrid): Thresholding at average of pretreatment scan CT V (airway segmentation)	SBRT	5-fraction Functional Lung Avoidance (FLA) fD_mean_ ↓ 1.5 fV13.5 ↓ 3.6 5-fraction Functionally Weighted Airway Sparing (FWAS) fD_mean_ ↓ 0.1 fV13.5 ↑ 0.1 5-fraction FLA + FWAS fD_mean_ ↓ 1.5 fV13.5 ↓ 3.7 3-fraction FLA fD_mean_ ↓ 1.5 fV11.4 ↓ 7.3 3-fraction FWAS fD_mean_ ↓ 0.2 fV13.5 ↓ 1.6 3-fraction FLA + FWAS fD_mean_ ↓ 2.4 fV13.5 ↓ 7.6 Individual-parameter significance not reported. Overall ventilation preservation, compared with conventional plan, was found to be significant.	Significance not reported 5-fraction FLA D_mean_ ↓ 1.2 V13.5 ↓ 3.1 5-fraction FWAS V13.5 ↓ 0.2 5-fraction FLA + FWAS D_mean_ ↓ 1.1 V13.5 ↓ 2.9 3-fraction FLA D_mean_ ↓ 1.8 V11.4 ↓ 4.7 3-fraction FLA + FWAS D_mean_ ↓ 1.7 V13.5 ↓ 5
Vinogradskiy^119^	67	65 (med)	All NSCLC	CT V (HU): 15% of max	IMRT	fMLD ↓ 1.3* fV5 ↓ 3.4* fV10 ↓ 6.4* fV20 ↓ 3.5* fV30 ↓ 1.8*	PTV coverage ↓ 0.8* MLD ↓ 0.7* V20 ↓ 2* Cord max ↑ 1.4* Mean esophagus ↓ 0.7*
Wang^113^	38	61 (med)	NSCLC	SPECT Q: 30% of max	IMRT	fV10 ↓ 5.21* fV15 ↓ 4.84* fV20 ↓ 4.25* fV25 ↓ 2.84* fV30 ↓ 2.38* fV35 ↓ 1.37* fV40 ↑ 0.43	No significant differences
Wang^115^	16	55 (med)	All NSCLC	CT V (Jacobian): Top 30%	IMRT: Equally spaced beams IMRT: Manual beams	fV5 ↓ 1.8* (equal) fV5 ↓ 1 (manual) fV10 ↓ 5.4* (equal) fV10 ↓ 1.1 (manual) fV20 ↓ 5.2* (equal) fV20 ↓ 1.1 (manual) fV30 ↓ 0.3 (equal) fV30 ↓ 1.5 (manual)	No significant difference
Waxweiler^118^	25	N/A	All lung cancer	CT V (HU): Maximum 15% reduction per lung third, weighted	IMRT	Thresholded fMLD ↓ 2.8* fV5 ↓ 13.7* fV10 ↓ 14.9* fV20 ↓ 5.6* fV30 ↓ 2.9* fV40 ↓ 1.6* Weighted fMLD ↓ 2.1* fV5 ↓ 11.3* fV10 ↓ 14.9* fV20 ↓ 5* fV30 ↓ 2	PTV max ↑ 1.3* HI ↓ 0.03* MLD ↓ 1.4* V20 ↓ 1.9* Cord max ↑ 3.6*	
Yamamoto^145^	1	N/A	NSCLC	CT V (HU): Weighted	IMRT	Single-patient data fV20 ↓ 5.1 fMLD ↓ 0.2	Single-patient data PTV homogeneity ↑ 1.6 V20 ↓ 4.5 V5 ↑ 10.6 MLD ↑ 0.2 Cord max ↑ 2.1 Mean esophagus ↑ 1.4
Yamamoto^109,†^	15	75.1 (mean)	All NSCLC	CT V (Jacobian): Thresholded into 3 equal volumes (unspecified)	VMAT IMRT	High-functioning lung fMLD ↓ 1.7425* (IMRT) fMLD ↓ 1.972* (VMAT) fV5 ↓ 2.09* (IMRT) fV5 ↓ 2.11* (VMAT) fV20 ↓ 3.37* (IMRT) fV20 ↓ 2.89* (VMAT) fV30 ↓ 2.8* (IMRT) fV30 ↓ 2.71* (VMAT) fV40 ↓ 2.54* (IMRT) fV40 ↓ 2.87* (VMAT) fV50 ↓ 2.32* (IMRT) fV50 ↓ 2.57* (VMAT) Moderate-functioning lung fMLD ↓* (IMRT) fMLD ↓ (VMAT) fV20 ↓ (IMRT) fV20 ↑ (VMAT) Low-functioning lung fMLD ↑ (VMAT) fV20 ↑ (IMRT) fV20 ↑ (VMAT)	MLD ↓ 0.7* (IMRT) PTV mean ↑ 2.1* (IMRT) PTV mean ↑ 1.8* (VMAT) CI ↓ 0.05* (IMRT) CI ↓ 0.03* (VMAT) HI ↑ 0.07* (IMRT) HI ↑ 0.07* (VMAT)
Yamamoto^148^	14	74 (med)	All NSCLC	CT V (elastic): Weighted	IMRT	fMLD ↓ 0.5* fV10 ↓ 2.2* fV20 ↓ 2.6* fV30 ↓ 1*	No significant differences
Yaremko^133^	27	65.7 (mean)	NSCLC	He-MRI V: Automated threshold into 3 regions	VMAT	All ventilated lung fV5 ↓ 3.5* fV10 ↓ 3.9* fV20 ↓ 2.9* fMLD ↓ 1* High-ventilated lung fV5 ↓ 4* fV10 ↓ 4.3* fV20 ↓ 4.4* fMLD ↓ 1.2*	PTV V95 ↓ 6* PTV max ↑ 0.9* V5 ↓ 3* V10 ↓ 3.3* V20 ↓ 2.4* MLD ↓ 0.8* Heart V40 ↑ 0.8* Mean esophagus ↓ 0.4*
Yaremko^107^	21	69 (med)	NSCLC	CT V (HU): 90th percentile	IMRT	Significance not reported. All functional dose-volume parameters improved in functional planning. VENT 1: Functional only fMLD ↓ 5.8 VENT 2: Functional with increased priority on PTV coverage fMLD ↓ 2.4	Significance not reported. PTV coverage was only sufficient for 11 patients in VENT 1 but all in VENT 2. VENT 1: Functional only HI ↓ 0.001 PTV 95% isodose ↑ 0.2 VENT2: Functional with increased priority on PTV coverage HI ↓ 0.193 PTV 95% isodiose ↓ 8.6
Yin^131^	10	N/A	NSCLC	SPECT Q: Visual thresholding	IMRT 3DCRT	fV5 ↓ (3DCRT) fV5 ↓* (IMRT) fV10 ↓* (IMRT, 3DCRT) fV20 ↓* (IMRT, 3DCRT) fV30 ↓* (3DCRT) fV40 ↓* (IMRT, 3DCRT) fMLD ↓* (IMRT, 3DCRT)	PTV V66 ↑* (IMRT, 3DCRT)

Anatomic plans were optimized without incorporating functional lung information, whereas functional-avoidance plans included this information.

*Abbreviations:* 3DCRT = 3-dimensional conformal radiation therapy; BTV = biologic tumor volume; CT = computed tomography; CTV = clinical target volume; FLI = functional lung imaging; fMLD = mean dose to functional lung; fVx = dose parameter (x) to functional lung, defined as in the modality column; FWAS = functionally weighted airway sparing; Ga = gallium; He = helium; HI = homogeneity index; HU = Hounsfield unit; IMPT = intensity modulated proton therapy; IMRT = intensity modulated radiation therapy; MLD = mean dose to non-PTV lung; MRI = magnetic resonance imaging; NSCLC = non-small cell lung cancer; OAR = organs at risk; PET = positron emission tomography; PTV = planning target volume; Q = perfusion; RBE = relative biological effectiveness; RT = radiation therapy; SBRT = stereotactic body radiation therapy; SCLC = small cell lung cancer; SPECT = single-photon emission computed tomography; SUV = standardized update volume; V/VENT = ventilation; VMAT = volumetric modulated arc therapy; Xe = xenon.

* Statistically significant.

† Data acquired via email.

**Table 2:** Using functional lung information to predict RILT

Study first author	N	Age	Cancer type	FLI modality: Definition	RT modality	RILT predicted	Best prediction outcome using functional parameters	Model used to make best prediction	Improvement in prediction compared with nonfunctional information
Bin^143^	217	60.8 (mean)	169 lung 48 esophageal	CT V (HU): Weighting	N/A	Grade 2+ RP	AUC: 0.874 (95% CI, 0.871-0.877)	Dual-omics model, combining radiomics with deep learning	N/A
Dhami^84^	20	67.5 (med)	15 NSCLC 2 SCLC 3 other	SPECT Q: Thresholding from 5%-95% of max, in 5% increments	3DCRT IMRT/VMAT PBT SBRT	Grade 2+ RP	Sensitivity: 100% Specificity: 81.25% *P* = .008	Cutoff at 13.3 Gy, using perfused mean lung dose (pMLD) at the 70th percentile threshold	No (univariate) Yes (bivariate)
Ding^73^	40	N/A	All NSCLC	SPECT Q: Thresholding from 10%-60% of max, in 10% increments Weighting	3DCRT IMRT	All grades RP	Thresholded AUC: 0.928 (95% CI, 0.842-1.013) Sensitivity: 90.9% Specificity: 86.2% Accuracy: 87.5%	Cutoff at 20% of maximum, for fV20, was the most predictive	N/A
Dougherty^86^	31	64 (med)	26 NSCLC 5 SCLC	CT V (HU): 15% of max, with further processing	VMAT IMPT	Grade 2+ RP Grade 3+ RP	Grade 2+: IMPT: NTCP ↓ 5.7% VMAT: NTCP ↓ 6.2% Grade 3+: IMPT: NTCP ↓ 2.4% VMAT: NTCP ↓ 3.4%	NTCP model from Faught et al^89,90^	Yes
Farr^152^	71	67 (med)	All NSCLC	SPECT Q: 20%, 40%, 60%, and 80% of max	IMRT SBRT	Grade 3+ RP	Baseline SPECT Q: AUC: 0.79 (95% CI, 0.68-0.91) Sensitivity: 72% Specificity: 70% Odds ratio: 7.8 Odds ratio with GTV: 9.2 Post-RT SPECT Q: AUC: 0.8 (95% CI, 0.62-0.94)	Baseline: Perfusion defect score cutoff Post-RT: Difference in defect score cutoff	N/A
Farr^72^	45	67 (median)	All NSCLC	SPECT Q: 20%, 40%, 60%, and 80% of max	IMRT SBRT	Symptomatic RP	Spearman’s r_s_ = 0.4, *P* = .02 Relative risk estimate: 3.6 (95% CI, 1.1-12)	Correlation analysis for r_s_ is for perfusion reduction in 21-40 Gy bin. Relative risk estimate reflects all reductions.	N/A
Farr^121^	58	67 (median)	All NSCLC	SPECT Q: Thresholding in 10% increments from 20th to 80th percentiles, followed by weighting	IMRT	Grade 2+ RP	Threshold odds ratio 1.53, *P* < .01 AUC = 0.81 (95% CI, 0.7-0.93) Weighting Odds ratio 1.4, *P* < .01 AUC = 0.78 (95% CI, 0.66-0.91)	Best for OR both thresholded and weighted models was found when using fMLD. Best for AUC for both thresholded and weighted models was found when using fV30	Yes
Faught^89^	70	N/A	All NSCLC	CT V (HU): 15% below average lung function	IMRT 3DCRT	Grade 2+ RP Grade 3+ RP	Grade 2+: AUC: 0.723, *P* < .01 NTCP ↓ 8%* (univariate) NTCP ↓ 10.4% (bivariate) Grade 3+: AUC: 0.674, *P* = .13 NTCP ↓ 4.8%* (univariate) NTCP ↓ 4.7% (bivariate)	Grade 2+: Best AUC uses fMLD Best univariate NTCP reduction uses fV10 Best bivariate NTCP reduction uses fV10 and V10 Grade 3+: Best AUC uses fV20 Best univariate NTCP reduction uses fV30 Best bivariate NTCP reduction uses fV10 and V10	N/A
Faught^90^	70	N/A	All NSCLC	CT V (HU): Thresholding from 5th to 95th percentile in increments of 5; nonlinear weighting	N/A	Grade 2+ RP Grade 3+ RP	Grade 2+: AUC: 0.73, *P* < .01 (threshold) AUC: 0.74, *P* < .01 (weighting) Grade 3+: AUC: 0.7, *P* = .033 (threshold) AUC: 0.67, *P* < .03 (weighting) NTCP 10% reduction: 17.2% NTCP 20% reduction: 30.2%	Grade 2+: Threshold best model used fV20 at 86th percentile or fMLD at 69th percentile. Sigmoid best model used amount of functional lung receiving ≥ 20 Gy (F20). Grade 3+: Threshold best model used fV20 at 85th percentile. Sigmoid best model used F20. Sigmoid NTCP 10% reduction requires a 17.2% reduction in F20 and a 20% reduction requires a 30.2% reduction in F20.	Yes
Gayed^174^	50	67.6 (mean)	All lung cancer	Planar Q: Lung perfusion score	IMRT 3DCRT Proton	Grade 2+ pulmonary complications (O_2_ dependence, respiratory failure, dyspnea, etc)	Lung perfusion score higher in patients with pulmonary complications, *P* = .01. Odds ratio = 1.6 (95% CI, 1.07-2.39) Odds ratio = 3.25 (95% CI, 1.37-7.70) (multivariate) AUC = 0.7	Lung perfusion score rates the perfusion defects seen in perfusion imaging	N/A
Hodge^135^	1	N/A	NSCLC	He-MRI V: Automated threshold	IMRT	Grade 3+ RP	Predicted risk of grade 3+ RP was 4% with functional planning	NTCP model assessing damage to individual functional subunits^179,180^	Yes
Hoover^144^	26	N/A	20 NSCLC 6 SCLC	SPECT V/Q: Weighting	N/A	Grade 2+ RP	AUC: 0.74 (ventilation) (95% CI, 0.54-0.94) AUC: 0.74 (perfusion) (95% CI, 0.54-0.93) Significant correlations found with increase in functional parameters and RP	N/A	Yes
Huang^140^	244	N/A	All lung cancer	CT V (HU): Weighting	IMRT VMAT	Grade 3+ RP	AUC: 0.77 Sensitivity: 0.71 Specificity: 0.76	Fully connected Convolutional neural network (CNN)	Yes
Huang^136^	36	66 (med)	All NSCLC	CT V (Xe-enhanced): >15 HU	VMAT IMRT	Grade 2+ RP	Relative risk reduction compared with anatomic plan: 30%, *P* < .001	Varian Eclipse’s biologic evaluations, using parameters from Seppenwoolde et al^181^	Yes
Kanai^96^	40	77 (med)	Thoracic cancers	CT V (HU): Thresholds from 5th to 95th percentiles	SBRT	Grade 2+ RP	AUC: 0.57	Best AUC found for fV30 at the 25th percentile threshold. Not statistically significant.	Yes
Kocak^141^	182	N/A	167 NSCLC 15 SCLC	SPECT Q: Weighting	N/A	Grade 2+ RP	One-tailed Fisher’s exact *P* = .03 on original data set, *P* = .33 and .41 on other data sets AUC: 0.65 (bivariate, Duke) AUC: 0.72 (univariate/bivariate, Netherlands cancer institute (NKI))	Model predicts high risk if a patient has MLD ≥ 25 Gy and pre-RT DLCO less than (Overall perfusion-weighted response parameter (OpRP) + 38) AUC for Duke data set was OpRP and FEV_1_ AUC for NKI was OpRP or OpRP and DLCO or OpRP and FEV_1_	Variable depending on data set
Lan^50^	37	61 (mean)	37 NSCLC	CT V (density change): Thresholded at 20%, 40%, 60% 80% of max; weighted	N/A	Grade 2+ radiation fibrosis	Lung consolidation: AUC: 0.66 (weighted) AUC: 0.65 (threshold) Volume loss: AUC: 0.71 (weighted) AUC: 0.75 (threshold) Statistically significant decrease in fV20, fV30, and fV40 for 60% thresholded parameters, for patients without volume loss Airway dilation: AUC: 0.8 (weighted) AUC: 0.85 (threshold) Statistically significant decrease in all thresholded functional parameters (60% threshold) and all but fV40 weighted parameters, for patients without airway dilation compared with those with	Lung consolidation weighting model used fV20, and threshold used fV30 at a threshold of 20% Volume loss model used fV30 or fV40 for weighting and 40% threshold fV40, 60% threshold fV40, or 80% threshold fV40 for thresholded method Airway dilation model used fV20 for weighting and fV20 at 40% threshold for thresholded method	Yes
Lee^79^	28	70.5 (med)	14 NSCLC 5 SCLC 5 Locally recurrent 4 Lung mets	SPECT Q: Threshold into 7 equidistant bins; 70% of max threshold used for dose-function parameters	VMAT proton IMRT SBRT 3DCRT	Grade 2+ RP	r_s_ = 0.94 AUC: 0.87, *P* =.011 pMLD cutoff 13.2 Gy EQD2: Sensitivity 100%; specificity 74%	Best functional Spearman coefficient was for fV20 using perfusion Best AUC was for pF20 (perfusion-weighted)	No
Li^130^	126	61 (med)	All lung cancer	CT Q: Model from Ren et al^15^	IMRT	Grade 2+ RP	AUC: 0.862 (95% CI, 0.851-0.871)	Dual radiomics and perfusion image-based model	Yes
Li^97^	17	67 (med)	All NSCLC	CT V (Jacobian): Top 10%, 20%, 30%, 40%, and 50% of max (planning); weighting (dose-function parameter calculation)	IMRT	Grade 2+ RP	Previous NTCP model successfully predicted which population would have statistically significant improvements in functional parameters due to avoidance planning compared with anatomic plans. For this population only: 50% thresholded fV5 ↓, fV10 ↓, fV20 ↓, fMLD ↓, V5 ↓, V10 ↓. 40% thresholded fv5 ↓, fV10 ↓, fV20 ↓, fMLD ↓, V5 ↓. 40% thresholded fV5 ↓, fV20 ↓, fMLD ↓ This population also had: 50% thresholded PTV HI ↑, Cord Max ↑. 40% thresholded PTV HI ↑, cord max ↑. 30% thresholded HI max ↑, cord max ↑	NTCP model^89^	Yes
Lind^142^	162	59 (mean)	118 Lung 20 Breast 17 Lymphoma 8 Other	SPECT Q: Weighting	N/A	Grade 2+ RP: Minimum 6 months follow-up	AUC: 0.62 (bivariate) AUC: 0.79 (bivariate with conditions) AUC: 0.83 (trivariate with conditions)	Bivariate: DLCO and overall response parameter (ORP) Bivariate with conditions: DLCO > 40; Mean perfused lung dose (MPLD) and DLCO Trivariate with conditions: DLCO > 40; DLCO, FEV_1_, and MPLD or ORP	Yes
Marks^182^	50	N/A	67 Lung 17 Breast 12 Lymphoma 4 Other	SPECT Q: N/A	N/A	RT-related pulmonary symptoms	NTCPs based on functional parameters provided no additional predictive value	N/A	No
O’Reilly^105^	74	N/A	All NSCLC	CT V (Jacobian): Top 6%, 45%, and 60% of max	Photon (unspecified) proton	Grade 2+ RP	AUC: 0.9 (photon) (95% CI, 0.74-0.98) AUC: 0.74 (proton) (95% CI, 0.53-0.89)	Best AUC used fMLD in highly ventilated lung	Yes
Otsuka^101^	40	66 (med)	All thoracic cancer	CT V: Threshold at percentile regions from 0-100, in increments of 10	SBRT other photon	Grade 2+ RP	AUC: 0.843 (95% CI, 0.732-0.954) For all functional parameters, grade 2+ RP had higher values than grade 1 RP	Best AUC used fV5 to low-ventilated region (0-30th percentiles)	N/A
Owen^106^	88	N/A	All NSCLC	SPECT V/Q: Percentile thresholds from 10-90, in increments of 10; analogous percentile thresholds of lowest function	3DCRT VMAT	Grade 2+ RP or clinical RF	Odds ratio: 0.05, *P* = .5 (Q) (95% CI, 0.01-0.91) Odds ratio: 0.06, *P* = .02 (V) (95% CI, 0.00-0.64) Odds ratio: 1.19, *P* = .006 (V and Q)	Best for Q was normalized fV20 in ipsilateral lung. Best for V was normalized fV20 in both lungs. Best for V and Q combined was the low-function volume receiving ≥20 Gy	No
Seppenwoolde^181^	382	N/A	274 NSCLC 66 Lymphoma 42 Breast	SPECT Q: NA	N/A	Grade 2+ RP Grade 2+ RP	Predictive; specific performance regarding quality of fit not specified. Log-likelihood of −119.4.	Sigmoid dose-effect relation fitted through SPECT Q	No
Sharifi^78^	30	N/A	All NSCLC	CT V (Jacobian) CT V (volume): Threshold at 95% of max and weighting	N/A	Grade 2+ dyspnea	Jacobian AUC: 0.79, *P* = .2 Volume AUC: 0.8, *P* = .01	Best AUC for Jacobian used fV1 to fV5 Best AUC for volume used fV1 to fV5	N/A
Thomas^126^	39	63 (med)	All NSCLC	SPECT Q: Radiomics, thresholding, and weighting separately each	IMRT VMAT PBT	Grade 2+ RP	No functional parameters were found to be significantly predictive of the endpoint.	N/A	No
Vinogradskiy^147^	96	N/A	All NSCLC	CT V (HU): Weighting	3DCRT IMRT	Grade 3+ RP	AUC: 0.62, *P* = .093	Best AUC used fV20. None were significant.	Yes
Wang^99^	57	N/A	All NSCLC	SPECT Q: Threshold 30% of max followed by weighting	IMRT 3DCRT	Grade 2+ RP	RP rate stratification of 2.9%:43.5% AUC: 0.869, *P* = .0001 Sensitivity: 0.9091 Specificity: 0.7391	Best AUC used fV15	Yes
Wang^104^	57	N/A	All NSCLC	SPECT Q: Threshold 30% of max followed by weighting	3DCRT IMRT	Grade 2+ RILT	All functional parameters from fV5 to fV50 in 5 Gy increments were significantly higher in patients with RILT AUC:0.869, *P* = .001 (95%CI,0.764-0.973)	Best AUC used fV15; best AUC better than volume-based fV40	No

**Table 3:** Comparison of functional parameters, PTV, and OARs for varying RT modalities using functional avoidance planning

Study first author	N	Age	Cancer type	FLI modality: Definition	RT modality comparison	Significant changes in dose-function parameters	Plan quality: Significant results
Christian^83^	6	N/A	All NSCLC	SPECT Q: Visual threshholding into 2 groups; weighting	Coplanar Noncoplanar	No significant changes	No significant changes
Dougherty^86^	31	64 (med)	26 NSCLC 5 SCLC	CT V (HU): 15% of max, with further processing	IMPT IMPT (dose-escalated) VMAT	IMPT changes vs VMAT fMLD ↓ 7.18* fV20 ↓ 9.18* fV30 ↓ 4.41* IMPT changes vs dose-escalated fV20 ↓ 1.37* fV30 ↓ 1.03*	IMPT reductions vs VMAT MLD ↓ 6.5* V20 ↓ 8.25* Mean esophagus ↓ 4.54* Mean heart ↓ 7.11* Cord max ↓ 23.34*
Farr^87^	15	71 (med)	12 NSCLC 1 SCLC 2 other	SPECT Q: 20%, 40%, 60%, and 80% of max	IMRT VMAT 3DCRT	3DCRT produced no statistically significant reductions or increases to functional parameters, aside from fV20 at the 40% threshold. VMAT produced statistically significant reductions in fV20 at the 20% threshold, fMLD and fV20 at the 40% threshold, fMLD and fV20 at the 60% threshold, and fMLD at the 80% threshold. IMRT produced statistically significant reductions for all functional parameters except fV5 for the 20% and 40% thresholds, and fV30 for the 60% and 80% thresholds.	Only the functional IMRT plan had statistically significant reductions in OAR-related parameters: V20 ↓ 2.02* V30 ↓ 1.03* Esophagus V60 ↓ 1.5* VMAT had a statistically significant increase in esophagus V60: Esophagus V60 ↑ 1.29*
Huang^92^	8	66 (med)	NSCLC	CT V (Jacobian): Thresholded into 2 groups (unspecified)	IMRT Double-scattering proton therapy (DSPT) IMPT	Only 2 IMRT plans were clinically acceptable. Both proton plans were superior in sparing low-dose regions. IMPT specifically did so while maintaining PTV coverage.	Significance not reported. V5 median IMRT > DSPT > IMPT V20 median IMRT = DSPT > IMPT MLD median IMRT > DSPT > IMPT Mean heart median IMRT > DSPT > IMPT Mean esophagus median IMRT = DSPT > IMPT Cord max IMRT > IMPT > DSPT
Ieko^93^	13	75.1 (mean)	All NSCLC	CT V (HU): 20th percentile	VMAT (SBRT) 3DCRT (SBRT) Proton (SBRT)	VMAT changes vs 3DCRT: fV10 ↓ 3.3* (threshold) fV10 ↓ 3* (weighting) Proton changes vs 3DCRT: fV5 ↓ 16* (threshold) fV5 ↓ 15.3* (weighting) fV10 ↓ 6.2* (threshold) fV10 ↓ 5.4* (weighting) fMLD ↓ 1.7* (threshold) fMLD ↓ 1.8* (weighting) Proton changes vs VMAT: fV5 ↓ 12.2* (threshold) fV5 ↓ 11.2* (weighting) fV10 ↓ 2.9* (threshold) fV10 ↓ 2.4* (weighting) fMLD ↓ 1.3* (threshold) fMLD ↓ 1.4* (weighting)	VMAT changes vs 3DCRT: V10 ↓ 3.2* contra-MLD ↑ 0.6* Cord max ↑ 3* Proton changes vs 3DCRT: PTV D_1_ ↓ 2.9* PTV D_99_ ↓ 1.4* MLD ↓ 1.7* V5 ↓ 15.7* V10 ↓ 6.2* contra-MLD ↓ 1.2* contra-V5 ↓ 8.9* contra-V20 ↓ 0.6* Cord max ↓ 9.1* Heart max ↓ 8.1* Proton changes vs VMAT: PTV D_1_ ↓ 2.3* PTV D_99_ ↓ 1* MLD ↓ 1.3* V5 ↓ 12.3* V10 ↓ 3* contra-MLD ↓ 1.8* contra-V5 ↓ 13* contra-V10 ↓ 0.7* Cord max ↓ 12.1* Heart max ↓ 8.5*
Kimura^69^	8	72.5 (med)	All lung cancer	CT (direct thresholding): > −860 HU	IMRT VMAT	IMRT changes VS VMAT: fV5 ↓* fV10 ↓* fV20 ↑*	IMRT changes vs VMAT: CI ↑ 0.64* Mean esophagus ↓ 0.7* PTV > 250 cc V5 ↓* PTV > 250 cc fV5 ↓*
Lavrenkov^94^	17	N/A	All NSCLC	SPECT Q: 60% of max	IMRT 3DCRT	IMRT changes vs 3DCRT stage III: fV20 ↓ 7.7* fMLD ↓ 2.7* IMRT changes vs 3DCRT nonuniform heterogeneous hypoperfusion: fV20 ↓ 11.5* fMLD ↓ 3.6*	IMRT changes vs 3DCRT stage III: PTV_90_/fV20 ↑ 2.1* IMRT changes vs 3DCRT nonuniform heterogeneous hypoperfusion: PTV_90_/fV20 ↑ 2.3*
Lavrenkov^95^	25	N/A	All NSCLC	SPECT Q: 60% of max	IMRT 3DCRT	IMRT changes vs 3DCRT stage III and nonuniform heterogeneous hypoperfusion: fV20 ↓ 10.6* fMLD ↓ 3.2*	IMRT changes vs 3DCRT stage III and nonuniform heterogeneous hypoperfusion: PTV_90_/fV20 ↑ 2.4*
Lee^21^	8	N/A	All NSCLC	SPECT Q: Threshold into 7 equidistant bins; 70% of max threshold used for dose-function parameters	VMAT proton VMAT and proton	(VMAT + proton) changes vs VMAT fMLD ↓* (median) (VMAT + proton) changes vs proton fMLD ↓* (median)	(VMAT + proton) changes vs VMAT PTV V60 ↑* (median) SUV_peak_ mean ↑* (median) MLD ↓* (median) V5 ↓* (median) V10 ↓* (median) V20 ↓* (median) Mean heart ↓* (median) Cord D_0.03_ ↓* (median) (VMAT + proton) changes vs proton PTV V60 ↑* (median) SUV_peak_ mean ↑* (median) MLD ↑* (median) Mean heart ↑* (median) Cord D_0.03_ ↑* (median)
McGuire^127^	5	N/A	All lung	SPECT Q: Threshold into 4 regions, with subsequent weighting	IMRT: 7 and 4 beams	Four beams vs 7 beams fV5 ↓ 8.6* fV13 ↓ 3.3* fV20 ↓ 1.4 fV30 ↑ 1.8	No significant differences
Mounessi^114^	13	N/A	NSCLC	SPECT Q: 30% of max	IMRT VMAT	Only IMRT gives a significant reduction in fV30 for localized hypoperfusion patients. Only VMAT gives a significant reduction in fV30 for diffuse hypoperfusion patients. Only IMRT gives a significant increase in V20 to nonfunctional lung. Only VMAT gives a significant increase to mean dose to nonfunctional lung.	No significant difference
Munawar^117^	10	N/A	NSCLC	SPECT V: Threshold at 50% and 70% of max; weighting	IMRT: 9 and 3 field	3-field change vs 9-field All patients No significant difference Group 1: Clinically acceptable fMLD ↓ 1.078* Group 2: Not acceptable No significant difference	3-field change vs 9-field All patients No significant difference Group 1: Clinically acceptable No significant difference Group 2: Not acceptable No significant difference
Tian^122^	10	N/A	NSCLC	SPECT Q: 4, 5, 7 beam: threshold (unspecified)	IMRT	4 beam changes vs 5 beam fV5 ↓ 10.28* fV30 ↑ 1.22* 4 beam changes vs 7 beam fV5 ↓ 15.24* fV20 ↑ 2.2* fV30 ↑ 1.6* fMLD ↓ 0.62* 5 beam changes vs 7 beam fV5 ↓ 4.96* fV20 ↑ 1.74* fV30 ↑ 0.48*	4 beam changes vs 5 beam CI ↓ 0.09* 4 beam changes vs 7 beam CI ↓ 0.1* 5 beam changes vs 7 beam HI ↑ 0.12*
Vicente^57^	12	64	All lung cancer	CT V (HU-Jacobian hybrid): Thresholding at average of pretreatment scan CT V (airway segmentation)	IMRT VMAT vs 3DCRT conventional plan All plans were SBRT	IMRT vs 3DCRT fD_mean_ ↓ 32% fV13.5 ↓ 39% VMAT vs 3DCRT fD_mean_ ↓ 14% fV13.5 ↓ 5%	Not reported
Wang^115^	16	55 (med)	All NSCLC	CT V (Jacobian): Top 30%	IMRT: Equally spaced beams IMRT: Manual beams	Manual change vs equal fV5 ↓ 13.2* fV10 ↓ 14.1* fV20 ↓4.4* fV30 ↓ 2	Manual change vs equal Mean cord ↓ 6.5* Max cord ↓ 4.4* Heart V60 ↑ 3.9*
Yamamoto^109^	15	75.1 (mean)	All NSCLC	CT V (Jacobian): Thresholded into 3 equal volumes (unspecified)	VMAT IMRT	Only IMRT had a significant decrease in fMLD and fV20 to moderately ventilated lung for the functional plan compared with anatomic.	Only IMRT had a significant reduction in mean lung dose in the functional plan compared with anatomic.
Yin^131^	10	N/A	NSCLC	SPECT Q: Visual thresholding	IMRT 3DCRT	IMRT changes vs 3DCRT fV5 ↑* fV40 ↓*	Significance not reported. IMRT changes vs 3DCRT PTV V66 ↑ Heart V30 ↑

*Abbreviations:* 3DCRT = 3-dimensional conformal radiation therapy; CT = computed tomography; FLI = functional lung imaging; fMLD = mean dose to functional lung; fVx = dose parameter (x) to functional lung, defined as in the modality column; HU = Hounsfield unit; IMPT = intensity modulated proton therapy; IMRT = intensity modulated radiation therapy; MLD = mean dose to non-PTV lung; Vx = dose parameter (x) to non-PTV lung; NSCLC = non-small cell lung cancer; OAR = organs at risk; PTV = planning target volume; Q = perfusion; RT = radiation therapy; SBRT = stereotactic body radiation therapy; SCLC = small cell lung cancer; SPECT = single-photon emission computed tomography; SUV = standardized update volume; V/VENT = ventilation; VMAT = volumetric modulated arc therapy.

* Statistically significant; anatomic plans not compared.

**Table 4:** Interventional trials involving functional lung avoidance

Study first author	N	Anatomic cohort?	FLI modality: Definition	RT modality	Technical results	Clinical outcomes	PTV and OAR, as compared with anatomic plan
Bucknell^82^	25	No (anatomic plans included for reference)	V/Q PET: 70th percentile threshold	VMAT with midtreatment adaptation	Perfusion No statistically significant difference in fV20 or fMLD fV5 ↓ 5.1* in the functional plan No statistically significant benefit to midtreatment functional adaptation. V, no statistically significant difference in fMLD fV20 ↓ 1.4* in the functional plan fV5 ↓ 5.0* in the functional plan No statistically significant benefit to midtreatment functional adaptation	The majority of patients with late-stage cancer benefitted from avoidance planning	Q, mean esophagus ↓ 0.6* V, no significant differences reported
Follacchio^76^	19	No (anatomic plans included for reference)	SPECT Q: 60% of maximum threshold	IMRT: Choice of functional or anatomic plan was made individually for each patient	The best plan for patients in all cases was judged to be the functional plan	Two cases of grade 2 RP were observed during follow-up. A significant correlation was found between perfusion score and early-onset RILT.	All functional plans were clinically acceptable.
Huang^136^	36	No (anatomic plans included for reference)	Xe CT V: Automated contouring	IMRT or VMAT	fV20 ↓ 1.7* in the functional plan fMLD ↓ 2.9* in the functional plan	4 grade 2+ RP cases diagnosed at follow-up, found to be statistically significantly lower than historical control. 1 case of grade 3 esophagitis. 50% of patients developed disease progression.	Maximum dose ↑ 1.5* V20 ↓ 2* MLD ↓ 1.1*
Miller^124^	56	No	CT V (HU-based): 15% of maximum threshold	N/A	Not reported	Avoidance planning reduces PFT results by less than standard anatomic plans compared with historical control.	Not reported
Thomas^126^	39	No	SPECT Q: N/A	19 IMRT/VMAT 20 Proton	Not reported	16 patients developed grade 2+ RP. COPD was the only significant predictor of grade 2+ RP development. No functional parameters were found to be significant.	Not reported
Thomas^102^	28 (8:20)	Yes	SPECT Q: 70% of maximum threshold	Anatomic plan: 6 IMRT/VMAT 2 proton functional plan: 12 IMRT/VMAT 2 proton	Medians reported. fMLD ↓ 7.9* in the functional cohort fV20 ↓ 12* in the functional cohort fV20 ↓ 23* in the functional cohort fV5 ↓ 24* in the functional cohort (all units EQD2)	Significant perfusion changes between cohorts were found cohort only in the 0-5 Gy dose bin. The functional cohort had increased cohort perfusion in this region.	No statistically significant differences found between cohorts.
Vinogradskiy^119^	67	No (anatomic plans included for reference)	CT V: 15% of maximum threshold	IMRT	fMLD ↓ 1.3* in the functional plan fV5 ↓ 3.4* in the functional plan fV10 ↓ 6.4* in the functional plan fV20 ↓3.5* in the functional fV30 ↓ 1.8* in the functional plan	10 grade 2+ RP events 33 grade 2+ esophagitis events 14 grade 2+ dyspnea events 13 grade 2+ cough events 14 grade 2+ fatigue events RP reduction was statistically significant compared with plan historical control.	PTV coverage ↓ 0.8* MLD ↓ 0.7* V20 ↓ 2* Cord max ↑ 1.4* Mean esophagus ↓ 0.7*
Yamamoto^148^	14	No (anatomic plans included for reference)	CT V: Weighted	IMRT with midtreatment adaptation	fMLD ↓ 0.7* in functional adapted plan fV10 ↓ 2.8* in functional adapted plan fV20 ↓ 2.5* in functional adapted plan fV30 ↓ 1.1* in functional adapted plan	Not reported	No significant differences found
Yaremko^133^	27 (11:16)	Yes	He-MRI: Threshold unspecifid	VMAT	No statistically significant reductions in dose-function parameters between cohorts.	No statistically significant differences in quality-of-life scores, RILT events, or disease progression statistics between cohorts.	No significant differences found between cohorts.

*Abbreviations:* COPD = chronic obstructive pulmonary disease; CT = computed tomography; EQD2 = equivalent dose in 2 Gy fractions; FLI = functional lung imaging; fMLD = mean dose to functional lung; fVx = dose parameter (x) to functional lung, defined as in the modality column; He = helium; HU = Hounsfield unit; IMRT = intensity modulated radiation therapy; MLD = mean dose to non-PTV lung; Vx = dose parameter (x) to non-PTV lung; MRI = magnetic resonance imaging; OAR = organs at risk; PET = positron emission tomography; PFT = pulmonary function test; PTV = planning target volume; Q = perfusion; RILT = radiation-induced lung toxicities; RP = radiation pneumonitis; RT = radiation therapy; SPECT = single-photon emission computed tomography; V = ventilation; VMAT = volumetric modulated arc therapy; Xe = xenon.

* Statistically significant.

## Data Availability

All data used are from published projects and are available publicly or upon request.
