# Supplementary material for: Incorporation of Functional Lung Imaging Into Radiation Therapy Planning in Patients With Lung Cancer: A Systematic Review and Meta-Analysis
Source: Int J Radiat Oncol Biol Phys. Author manuscript; Available in PMC 2024 Nov 21. (PMC11580018; doi:10.1016/j.ijrobp.2024.04.001)
Supplement: Sup3 [file NIHMS2033239-supplement-Sup3.pdf]

B1: Ventilation fV20

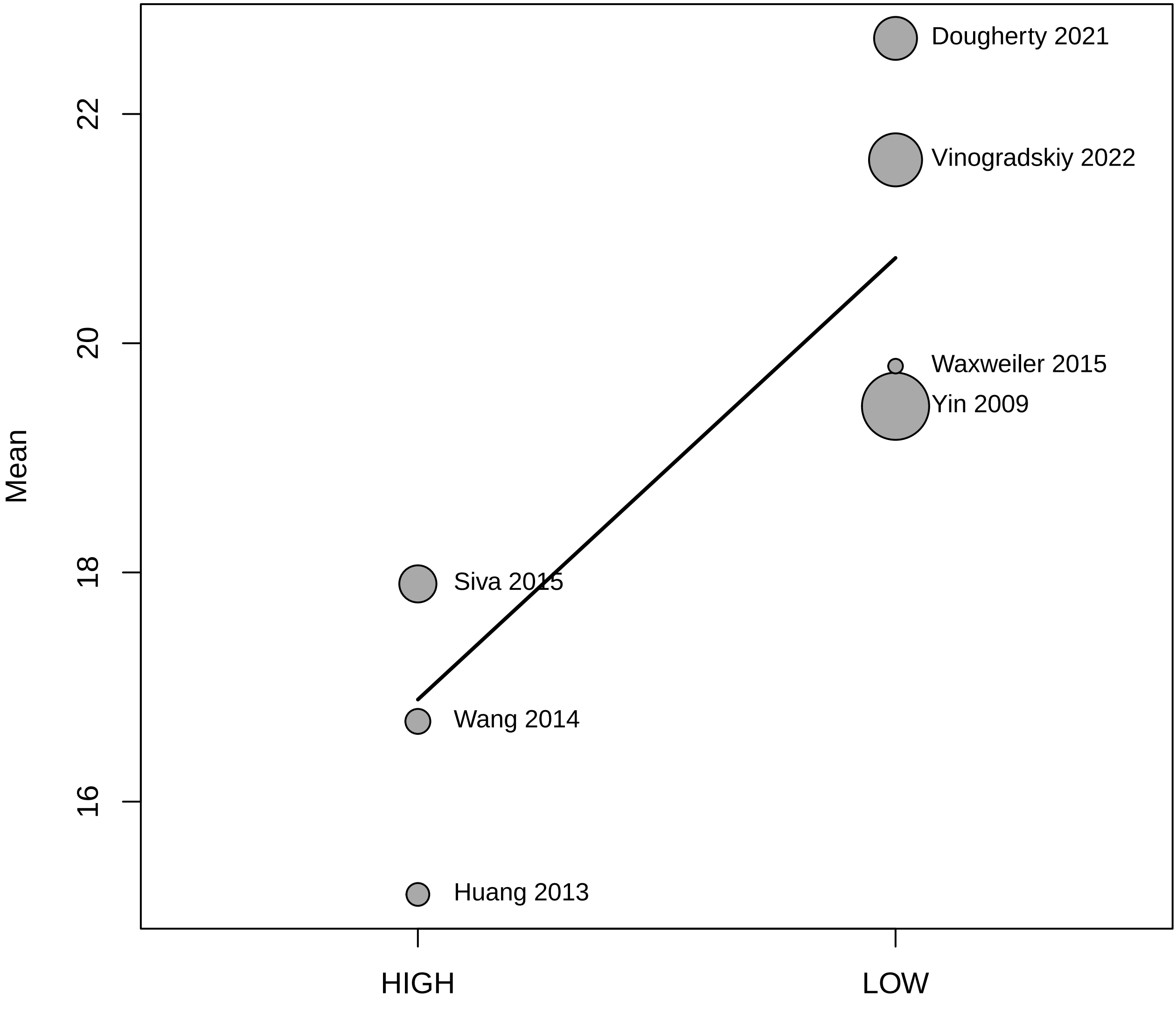

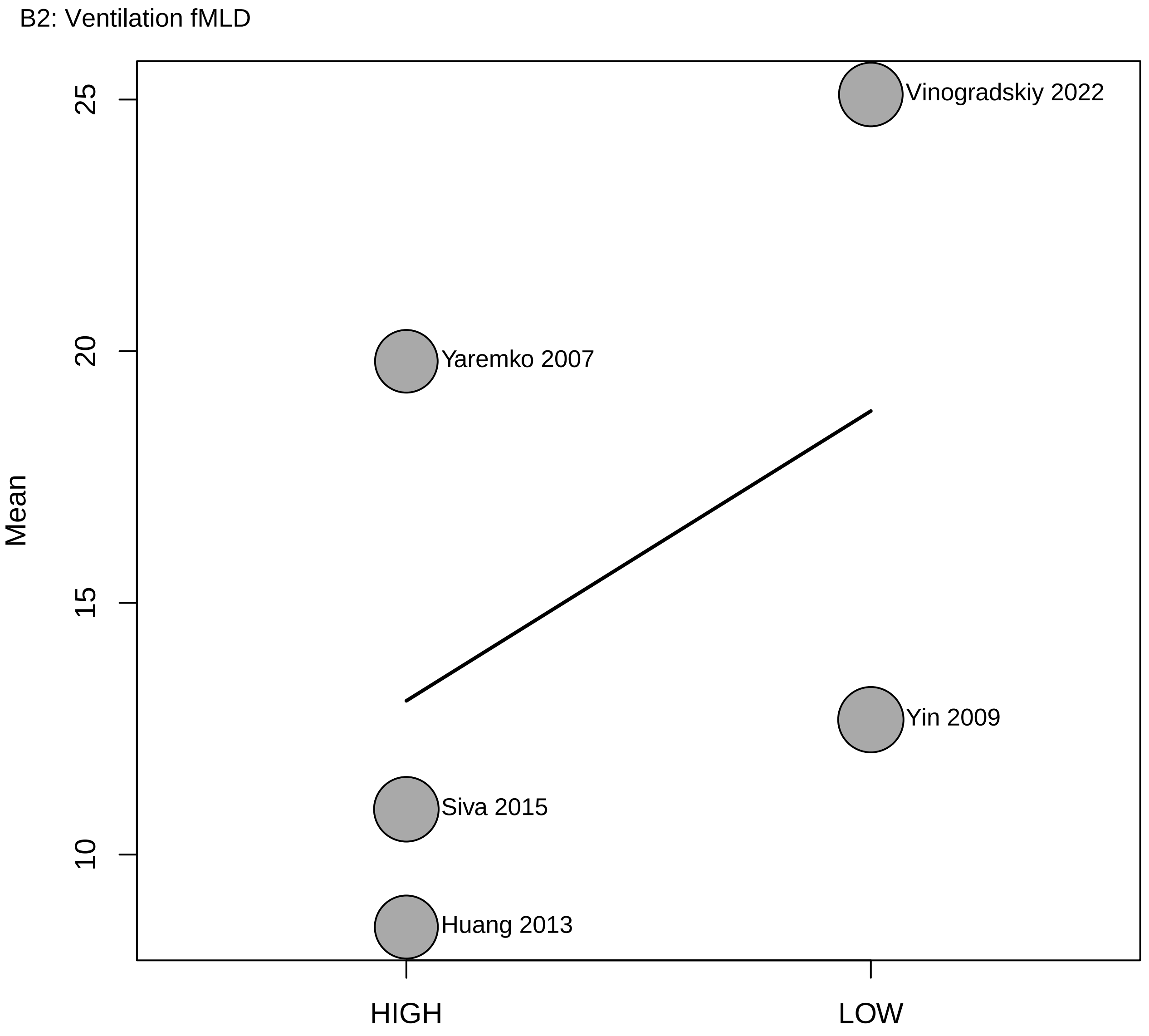

Supplementary Figure B: Meta-regression results. High-threshold definitions had a cutoff greater than or equal to 70% of maximum function, or the 70th percentile. Low-threshold definitions had a cutoff less than or equal to 30% of maximum function, or the 30th percentile. (B1) Ventilation fV20 (B2) Ventilation fMLD
