# Supplementary material for: Incorporation of Functional Lung Imaging Into Radiation Therapy Planning in Patients With Lung Cancer: A Systematic Review and Meta-Analysis
Source: Int J Radiat Oncol Biol Phys. Author manuscript; Available in PMC 2024 Nov 21. (PMC11580018; doi:10.1016/j.ijrobp.2024.04.001)
Supplement: Sup2 [file NIHMS2033239-supplement-Sup2.pdf]

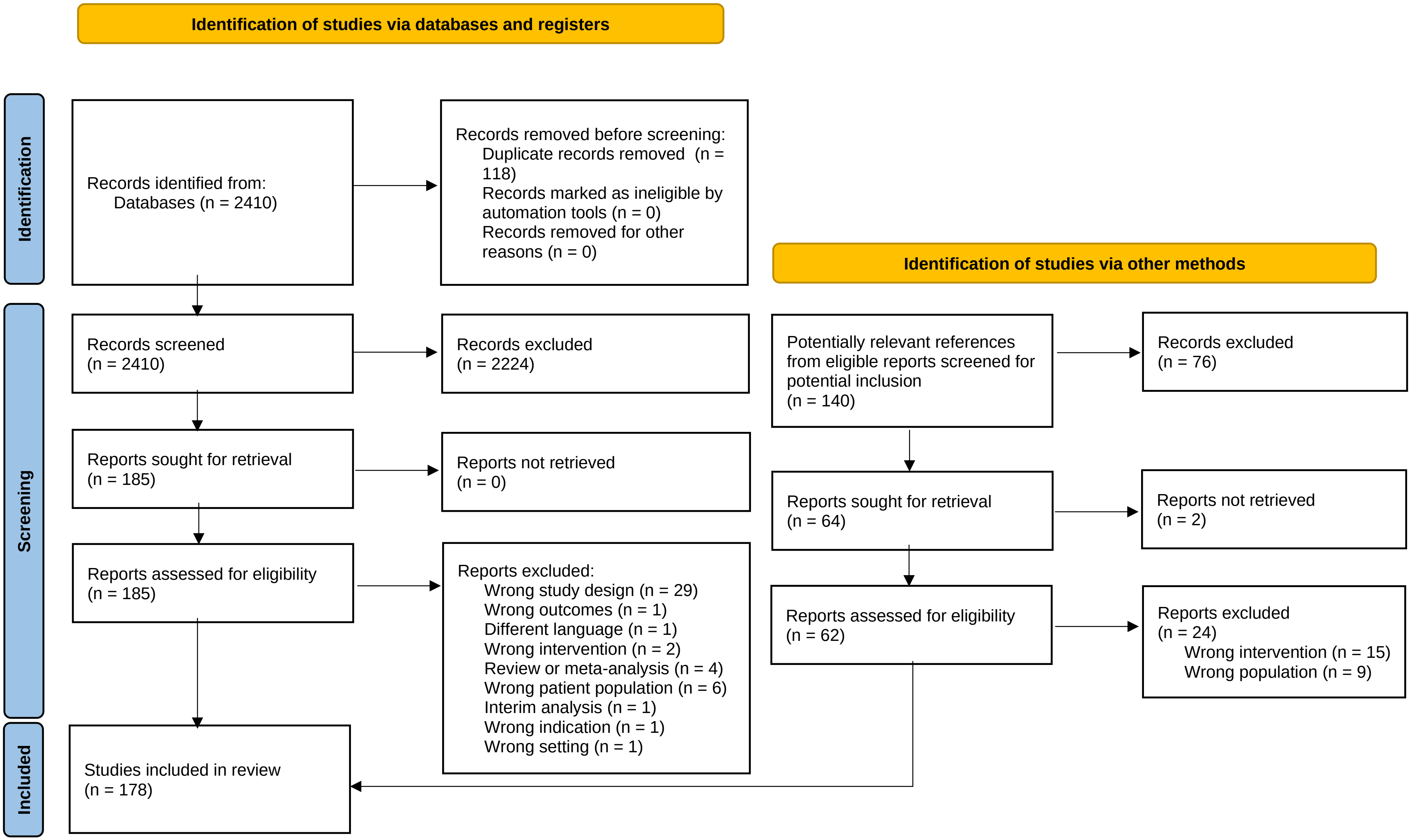
Identification of studies via databases and registers
Identification
Records identified from:
Databases (n = 2410)
Records removed before screening:
Duplicate records removed (n = 118)
Records marked as ineligible by automation tools (n = 0)
Records removed for other reasons (n = 0)
Identification of studies via other methods
Screening
Records screened
(n = 2410)
Records excluded
(n = 2224)
Potentially relevant references from eligible reports screened for potential inclusion
(n = 140)
Records excluded
(n = 76)
Reports sought for retrieval
(n = 185)
Reports not retrieved
(n = 0)
Reports sought for retrieval
(n = 64)
Reports not retrieved
(n = 2)
Reports assessed for eligibility
(n = 185)
Reports excluded:
Wrong study design (n = 29)
Wrong outcomes (n = 1)
Different language (n = 1)
Wrong intervention (n = 2)
Review or meta-analysis (n = 4)
Wrong patient population (n = 6)
Interim analysis (n = 1)
Wrong indication (n = 1)
Wrong setting (n = 1)
Reports assessed for eligibility
(n = 62)
Reports excluded
(n = 24)
Wrong intervention (n = 15)
Wrong population (n = 9)
Included
Studies included in review
(n = 178)
