# Supplementary material for: Incorporation of Functional Lung Imaging Into Radiation Therapy Planning in Patients With Lung Cancer: A Systematic Review and Meta-Analysis
Source: Int J Radiat Oncol Biol Phys. Author manuscript; Available in PMC 2024 Nov 21. (PMC11580018; doi:10.1016/j.ijrobp.2024.04.001)
Supplement: Sup1 [file NIHMS2033239-supplement-Sup1.pdf]

**Supplementary A: Search Strategy and Screening Criteria**

*EMBASE & Cochrane Library*

(exp lung/ or exp lung cancer/ or lung.mp.)

and

(radiotherapy.mp. or exp radiotherapy/ or radiation therapy.mp. or exp stereotactic body radiation therapy/ or exp stereotactic radiosurgery/ or stereotactic.mp. or radiosurgery.mp. or exp radiosurgery/)

and

(exp lung disease/ or exp toxicity/ or toxicity.mp. or Radiation Pneumonitis.mp. or exp radiation pneumonia/ or radiation injury.mp. or exp radiation injury/ or radiation injuries.mp. or lung injury.mp. or exp lung injury/ or radiation pneumonia.mp.)

and

(pulmonary ventilation.mp. or exp lung ventilation/ or pulmonary function*.mp. or lung spar*.mp. or exp lung function/ or functional lung.mp. or respiratory decline.mp. or exp respiratory failure/ or lung volume.mp. or exp lung volume/ or Lung Volume Measurements.mp. or exp ventilation-perfusion scan/ or Perfusion Imaging.mp. or exp scintigraphy/ or Ventilation-Perfusion Ratio.mp. or exp lung ventilation perfusion ratio/ or Respiratory Function.mp. or exp respiratory function/ or irradiated lung volume.mp.)

Limit to Human and English language and yr="1990 -Current"

*PubMed/Medline*

(lung[tw] OR lung neoplasms[mh])

AND

(radiotherapy[mh] OR radiation therapy[tw] OR radiotherapy[tw] OR stereotactic[tw] OR radiosurgery[mh])

AND

(Lung Diseases[mh] OR toxicity[tw] OR Radiation Pneumonitis[mh] OR radiation pneumonitis[tw] OR Radiation Pneumonia[tw] OR radiation injury[mh] OR radiation injuries[mh] OR lung injury[mh] OR toxicity[sh])

AND

(pulmonary ventilation[mh] OR pulmonary function*[tw] OR lung spar*[tw] OR functional lung[tw] OR respiratory declin*[tw] OR lung volume[tw] OR Lung Volume Measurements[mh] OR Ventilation-Perfusion Scan[mh] OR pulmonary ventilation[tw] OR Perfusion Imaging[mh] OR Ventilation-Perfusion Ratio[mh] OR Respiratory Function[tw] OR irradiated lung volume[tw])

Filters: English, Humans, from 1990 - 2023

*Screening Criteria: Title and Abstract*

INCLUSION

- FLI in the context of lung cancer
- FLI and RP in the same abstract
- Functional avoidance planning mentioned

EXCLUSION

- Protocol
- Review article
- Abstract only
- Pediatric populations for anything other than modalities
- Animal models for anything other than modalities
- Non-lung neoplasm exclusively, for anything other than scan methods
- PFT test with no mention of FLI

*Screening Criteria: Full-Text*

INCLUSION

1) Technical details of imaging techniques
2) Comparisons of imaging techniques
3) Location of functional lung
4) Methods of integration into RT plannin and delivery
5) Reduction of RT: functional lung ratio
6) Best RT techniques to combine with FLI
7) Relationship between functional lung irradiation and loss of function, or RILT develofpment
8) Use of FLI to quantify lung toxicity by way of pre- and post-treatment scans

EXCLUSION

1) Non-lung neoplasm for anything other than scan methods
2) Pediatric for anything other than scan methods
3) Review article

Regional perfusion not related to identifying functional lung

**Supplementary B: Extraction Headers**

Study Name

First Author

Study Date

N

Study Type

Time Point(s)

Population

RT Modality

FLI Modality

Modality

Technical Description

  Functional Lung Definition

  Functional Lung LocationModality Comparison

Modalities Compared

Comparator

Comparison

Treatment Planning

Imaging Modality

Description of RT Plan Creation

Comparisons: Methods of RT Delivery

Comparisons: Func VS Anat

Lung Function

Imaging Modality

Radiation

Function Metric

Dose-Response Model

Toxicity

Imaging Modality

Radiation

Toxicity Definition

Correlation with Toxicity

Metric of Risk

Predictive Model

Other Information

**Supplementary C: Statistical Methods for Analyses**

1. **Meta-analysis of mean difference**

Meta-analysis was conducted using functions in the R package *meta.*[193] Meta-analysis of mean difference between the anatomical and functional RT plans was performed using *metamean*() function and forest plot was used to display the results. Sensitivity analyses of mean difference were conducted using the same procedure.

When the sample size (n), mean difference and SD of the mean difference were available, these statistics were directly used as inputs for the meta-analysis. Alternatively, SD was calculated from standard error as SE x sqrt (n). When SE was not reported, the p-value of a two-sided paired-t test with (n-1) degrees of freedom at a significance level of 0.05 was used to calculate the t-statistic first, and then SD was derived as [mean difference / t-statistic x sqrt(n)]. When neither SE or p-value was reported, missing means were approximated using n, median and range, based on methods described in equation (7) in Luo et al.,[194] and missing standard deviations were approximated from n, median and range, based on equation (11) in Shi et al. [195]; both were implemented in the *metamean*() function.

Table 2 Studies included in the meta-analysis and sensitivity meta-analysis.

| Type | Study | If SD was not reported, how was it derived? |
|---|---|---|
| Ventilation fV20 | Ireland 2007[139] | reported |
| Ventilation fV20 | Yaremko 2007[107] | SD = SE x sqrt (n) |
| Ventilation fV20 | Yamamoto 2011[109] | reported |
| Ventilation fV20 | Wang 2014[115] | calculated using p-value of a paired t-test |
| Ventilation fV20 | Siva 2015[111] | SD = SE x sqrt (n) |
| Ventilation fV20 | Kadoya 2015[120] | calculated using p-value of a paired t-test |

**Additional references for statistical analysis**

**[193]** S Balduzzi, G Rücker and G. Schwarzer, How to perform a meta-analysis with R: A practical tutorial, Evid Based Ment Health, 22, 2019, 153–160.

**[194]** D Luo, X Wan, J Liu and T. Tong, Optimally estimating the sample mean from the sample size, median, mid-range, and/or mid-quartile range, Stat Methods Med Res, 27, 2016, 1785–1805.

**[195]** J Shi, D Luo, H Weng, et al., Optimally estimating the sample standard deviation from the five-number summary, Res Synth Methods, 11, 2020, 641–654.

| | | |
|---|---|---|
| Ventilation fV20 | Waxweiler 2017[118] | reported |
| Ventilation fV20 | Dougherty 2021[86] | reported |
| Ventilation fV20 | Feng 2021[39] | Missing SD was derived from n and range |
| Ventilation fV20 | Vinogradskiy 2022[119] | reported |
| Ventilation fV20 | Yaremko 2022[133] | reported |
| Ventilation fV20 | Ding 2022[85] | reported |
| Ventilation fV20 | Huang 2023[136] | calculated using p-value of a paired t-test |
| Perfusion fV20 | Christian 2005[83] | SD = SE x sqrt (n) |
| Perfusion fV20 | Shioyama 2007[110] | Missing mean and SD were derived from n, median and range |
| Perfusion fV20 | Agrawal 2012[137] | reported |
| Perfusion fV20 | Wang 2013[113] | calculated using t test statistic |
| Perfusion fV20 | Tian 2014[122] | reported |
| Perfusion fV20 | Siva 2015[111] | SD = SE x sqrt (n) |
| Perfusion fV20 | Farr 2019[87] | SD = SE x sqrt (n) |
| Perfusion fV20 | Mounessi 2020[114] | reported |
| Perfusion fV20 | Greco 2022[91] | calculated using p-value of a paired t-test |
| Perfusion fV20 | Lucia 2023[116] | Missing mean and SD were derived from n, median and range |
| Ventilation fMLD | Ireland 2007[139] | reported |
| Ventilation fMLD | Yamamoto 2011[109] | reported |
| Ventilation fMLD | Siva 2015[111] | SD = SE x sqrt (n) |
| Ventilation fMLD | Kadoya 2015[120] | calculated using p-value of a paired t-test |
| Ventilation fMLD | Waxweiler 2017[118] | reported |
| Ventilation fMLD | Feng 2021[39] | Missing SD was derived from n and range |

| | | |
|---|---|---|
| Ventilation fMLD | Vinogradskiy 2022[119] | reported |
| Ventilation fMLD | Yaremko 2022[133] | reported |
| Ventilation fMLD | Ding 2022[85] | reported |
| Ventilation fMLD | Huang 2023[136] | calculated using p-value of a paired t-test |
| perfusion fMLD | Christian 2005[83] | reported |
| perfusion fMLD | Shioyama 2007[110] | Missing mean and SD were derived from n, median and range |
| perfusion fMLD | Agrawal 2012[137] | reported |
| perfusion fMLD | Siva 2015[111] | SD = SE x sqrt (n) |
| perfusion fMLD | Farr 2019[87] | SD = SE x sqrt (n) |
| perfusion fMLD | Lucia 2023[116] | Missing mean and SD were derived from n, median and range |

## 2. Meta-regression analysis

To assess the relationship between the threshold and the mean difference, meta-regression was fitted using *metareg*() function and bubble plot was used to display the results.

Table 2 Studies included in the meta-regression

| Type | Study | If SD was not reported, how was it derived? |
|---|---|---|
| fV20-vent | Dougherty 2021[86] | reported |
| fV20-vent | Huang 2013[71] | reported |
| fV20-vent | Siva 2015[111] | reported |
| fV20-vent | Vinogradskiy 2022[119] | reported |

| | | |
|---|---|---|
| fV20-vent | Wang 2014[115] | reported |
| fV20-vent | Waxweiler 2017[118] | reported |
| fV20-vent | Yin 2009[131] | reported |
| fMLD-vent | Huang 2013[71] | reported |
| fMLD-vent | Siva 2015[111] | reported |
| fMLD-vent | Vinogradskiy 2022[119] | reported |
| fMLD-vent | Yaremko 2007[107] | SD = SE x sqrt (n) |
| fMLD-vent | Yin 2009[131] | reported |

## 3. Meta-analysis of AUC values for prediction of grade 2+ RP

To meta-analyze AUC values for prediction of grade 2+ RP, *metamean*() function was used and forest plot was displayed for each subgroup (anatomical, perfusion, or ventilation).

When SD was not reported, it was calculated as SE x sqrt (n) if the SE was reported. Alternatively, if the 95% confidence interval (CI) or the p-value was reported, SD was estimated based on normal approximation or a z-test.

Table 4 Studies included in the meta-analysis of the AUC for prediction of grade 2+ RP.

| Study | If SD was not reported, how was it derived? |
|---|---|
| Dhami 2017[84] | calculated based on normal approximation using reported CI |
| Farr 2015[121] | calculated based on normal approximation using reported CI |
| Hoover 2013[144] | calculated based on normal approximation using reported CI |
| Lee 2018[79] | SD = SE x sqrt(n) |

| | |
|---|---|
| Li 2022[58] | Reported |
| O'Rielly 2020[105] | calculated based on normal approximation using reported CI |
| Wang 2011[99] | calculated based on normal approximation using reported p-value |
| Bin 2021[143] | calculated based on normal approximation using reported CI |
| Faught 2017[89] | calculated based on normal approximation using reported p-value |
| Otsuka 2018[101] | calculated based on normal approximation using reported CI |

## 4. Meta-analysis of proportion of grade 2+ RP

The proportion of patients who had grade 2+ RP was meta-analyzed using *metaprop*() function and forest plot was used to display the results. All studies included in the meta-analysis reported the number of events and sample size, no approximation method was needed.

**Supplementary D: Exclusions from Relevant Statistical Analyses**

Below are lists of reasons for exclusion of all studies in each relevant table, from the relevant analysis. Patient-level exclusions from analyses are mentioned in the main text and not listed below.

*Meta-analysis and Sensitivity*

| **Reason for Exclusion** | **Studies** |
|---|---|
| Wrong FLI modality or definition | Doi et al., 2017[151]<br>Iqbal et al., 2023[81]<br>Kida et al., 2016[77]<br>Kimura et al., 2012[69]<br>Matrosic et al., 2021[65]<br>Matuszak et al., 2016[146]<br>McGuire et al., 2006[108]<br>McGuire et al., 2009[127]<br>Munawar et al., 2010[117]<br>Seppenwoolde et al., 2002[149]<br>St-Hilaire et al., 2011[150]<br>Yamamoto et al., 2016[145]<br>Yamamoto et al., 2018[148] |
| Statistical information not provided; author unable to provide data via email | Faught et al., 2017[90]<br>Huang et al., 2018[92]<br>Huang et al., 2013[71]<br>Lee et al., 2017[21] |

| | |
|---|---|
| Case study of one patient | Hodge et al., 2010[135]<br>Miften et al., 2004[125] |
| Extensively overlapping population | Siva et al., 2016[112] |

The sensitivity analysis was conducted with the same studies as the above meta-analysis

*Meta-Regression*

| **Reason for Exclusion** | **Studies** |
|---|---|
| Wrong FLI modality or definition | Doi et al., 2017[151]<br>Kimura et al., 2012[69]<br>Matrosic et al., 2021[65]<br>Matuszak et al., 2016[146]<br>McGuire et al., 2006[108]<br>McGuire et al., 2009[127]<br>Munawar et al., 2010[117]<br>Seppenwoolde et al., 2002[149]<br>St-Hilaire et al., 2011[150]<br>Yamamoto et al., 2016[145]<br>Yamamoto et al, 2018[148] |
| Statistical information not provided; author unable to provide data via email | Faught et al., 2017[89]<br>Kadoya et al., 2015[120]<br>Lee et al., 2017[21] |
| Case study of one patient | Hodge et al., 2010[135] |

| | |
|---|---|
| Extensively overlapping population with another study | Siva et al., 2016[112] |
| Improper threshold cutoff or multiple threshold optimization objectives | Agrawal et al., 2012[137]<br>Christian et al., 2005[83]<br>Ding et al., 2022[85]<br>Farr et al., 2019[87]<br>Feng et al., 2021[39]<br>Greco et al., 2022[91]<br>Huang et al., 2018[92]<br>Huang et al., 2023[136]<br>Ireland et al., 2007[139]<br>Iqbal et al., 2023[81]<br>Kida et al., 2016[77]<br>Lucia et al., 2023[116]<br>Miften et al., 2004[134]<br>Shioyama et al., 2007[110]<br>Tian et al., 2014[122]<br>Yamamoto et al., 2011[109]<br>Yaremko et al., 2022[133] |
| Perfusion study with no exclusion criteria, omitted due to lack of other datapoints | Wang 2013[113]<br>Mounessi 2020[114] |

*AUC Analysis*

| **Reason for Exclusion** | **Studies** |
|---|---|

| | |
|---|---|
| Statistical information not provided; author unable to provide data via email | Kanai et al., 2018[96]<br>Kocak et al., 2007[141]<br>Lind et al., 2002[142]<br>Wang et al., 2011[99] |
| Case study of one patient | Hodge et al., 2010[135] |
| Wrong toxicity-related outcome or descriptive statistic | Ding et al., 2018[73]<br>Dougherty et al., 2021[86]<br>Farr et al., 2015[72]<br>Farr et al., 2015[121]<br>Farr et al., 2015[152]<br>Follacchio et al., 2020[76]<br>Gayed et al., 2008[173]<br>Huang et al., 2021[140]<br>Huang et al., 2023[136]<br>Lan et al., 2016[50]<br>Li et al., 2023[97]<br>Marks et al., 1997[130]<br>Owen et al., 2021[106]<br>Sharifi et al., 2019[78]<br>Thomas et al., 2022[126]<br>Vinogradskiy et al., 2013[147]<br>Vinogradskiy et al., 2022[119]<br>Wang et al., 2012[104]<br>Wang et al., 2012[123]<br>Weller et al., 2019[100] |

| | |
|---|---|
| | Xiao et al., 2017[125]<br><br>Xiao et al., 2018[103]<br><br>Yaremko et al., 2022[133] |
| Excessively overlapping population | Faught et al., 2017[90] |

*Interventional Trials*

| **Reason for Exclusion** | **Studies** |
|---|---|
| No RP rate reported | Bucknell et al., 2023[82]<br><br>Miller et al., 2023[124]<br><br>Thomas et al., 2019[102]<br><br>Yamamoto et al., 2018[148] |
| Other (specified) | Thomas et al., 2022[126]: PBT confounding |
