# Supplementary material for: Incorporation of Functional Lung Imaging Into Radiation Therapy Planning in Patients With Lung Cancer: A Systematic Review and Meta-Analysis
Source: Int J Radiat Oncol Biol Phys. Author manuscript; Available in PMC 2024 Nov 21. (PMC11580018; doi:10.1016/j.ijrobp.2024.04.001)
Supplement: Sup4 [file NIHMS2033239-supplement-Sup4.pdf]

Supplementary Figure C: Sensitivity Analysis

(C1)

Study	Mean	SD	Total	Weight	Mean IV, Random, 95% CI
Ireland 2007	2.46	2.00	5	7.6%	2.46 [0.71; 4.21]
Yaremko 2007	3.75	5.50	21	5.4%	3.75 [1.40; 6.10]
Yamamoto 2011	3.37	3.30	15	7.9%	3.37 [1.70; 5.04]
Wang 2014	5.20	7.99	16	2.6%	5.20 [1.28; 9.12]
Siva 2015	0.30	9.03	20	2.5%	0.30 [−3.66; 4.26]
Kadoya 2015	3.20	8.44	22	3.0%	3.20 [−0.33; 6.73]
Waxweiler 2017	5.65	4.99	25	6.8%	5.65 [3.70; 7.61]
Dougherty 2021	4.18	2.82	31	11.3%	4.18 [3.19; 5.17]
Feng 2021	1.82	1.07	20	13.8%	1.82 [1.35; 2.29]
Vinogradskiy 2022	3.50	2.52	67	13.3%	3.50 [2.89; 4.10]
Yaremko 2022	3.00	0.80	27	14.3%	3.00 [2.70; 3.30]
Ding 2022	1.57	2.17	10	9.5%	1.57 [0.23; 2.92]
Huang 2023	2.70	13.85	36	2.0%	2.70 [−1.82; 7.22]
**Total (95% CI)**			**315**	**100.0%**	**3.10 [2.41; 3.79]**

Heterogeneity: $Tau^2 = 0.8429$; $Chi^2 = 46.15$, df = 12 ($P < 0.01$); $I^2 = 74\%$

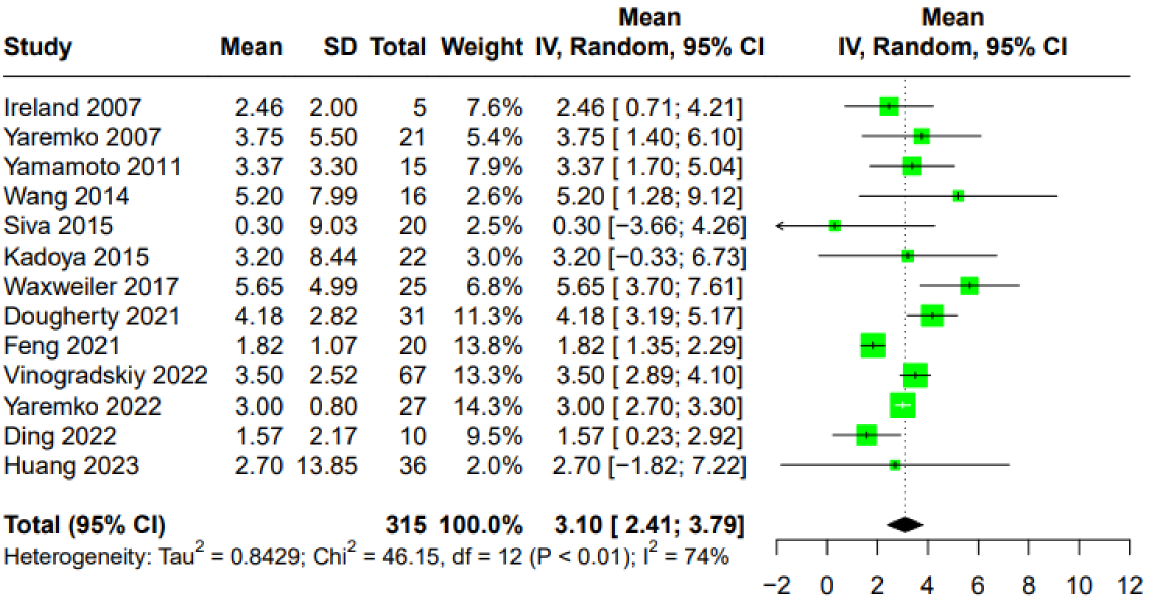

(C2)

Study	Mean	SD	Total	Weight	Mean IV, Random, 95% CI
Christian 2005	0.47	0.50	5	13.4%	0.47 [0.04; 0.91]
Shioyama 2007	9.06	7.48	14	7.5%	9.06 [5.14; 12.98]
Agrawal 2012	6.05	8.57	11	5.8%	6.05 [0.99; 11.11]
Wang 2013	4.25	4.85	39	12.1%	4.25 [2.73; 5.77]
Tian 2014	3.14	2.46	10	12.1%	3.14 [1.62; 4.66]
Siva 2015	3.80	7.78	20	8.4%	3.80 [0.39; 7.21]
Farr 2019	3.36	5.58	15	9.6%	3.36 [0.54; 6.18]
Mounessi 2020	2.21	2.21	13	12.6%	2.21 [1.01; 3.41]
Greco 2022	7.00	8.90	9	4.9%	7.00 [1.19; 12.81]
Lucia 2023	0.19	0.37	60	13.6%	0.19 [0.09; 0.28]
**Total (95% CI)**			**196**	**100.0%**	**3.28 [1.67; 4.88]**

Heterogeneity: $Tau^2 = 4.9588$; $Chi^2 = 91.87$, df = 9 ($P < 0.01$); $I^2 = 90\%$

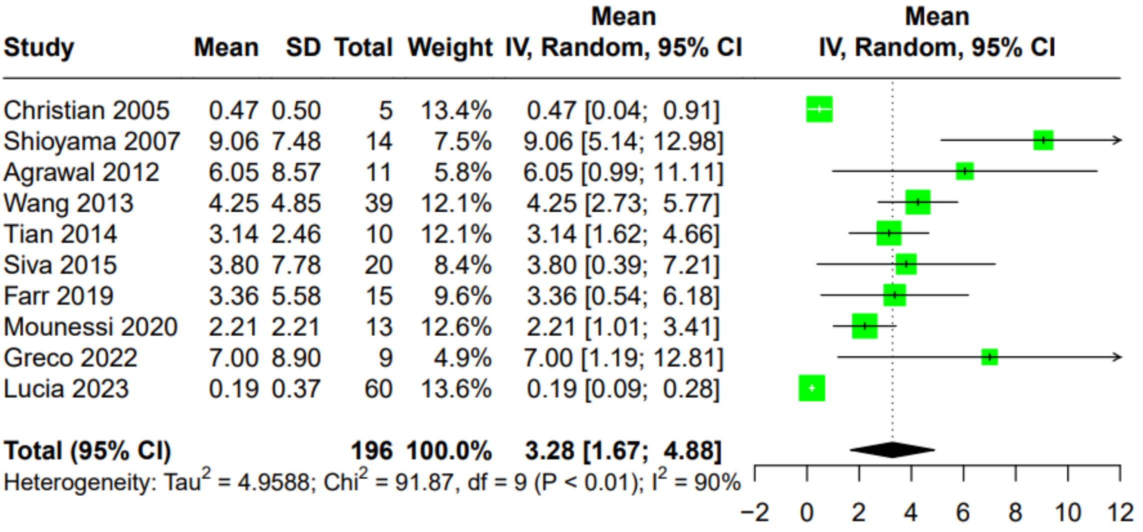

(C3)

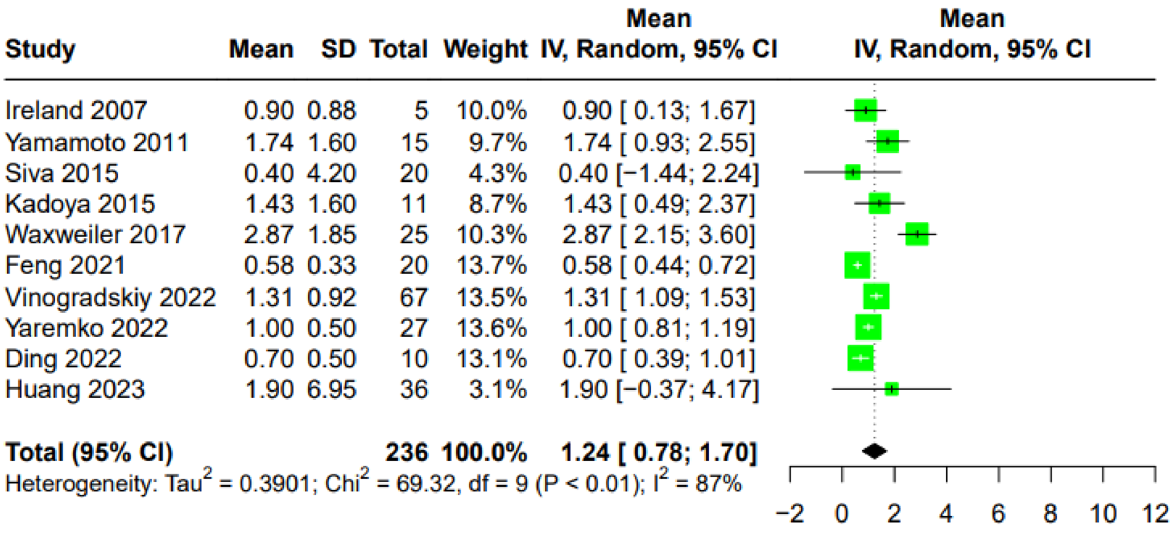

(C4)

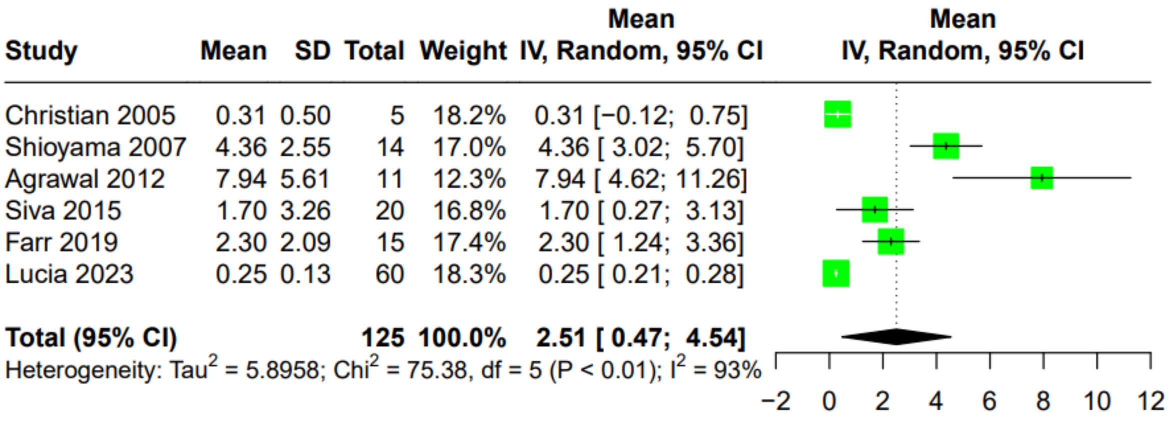

Supplementary Figure C: Sensitivity analysis of studies which reported multiple definitions of functional lung. Results are very similar to the original meta-analysis. (1): fV20-vent. Alternate FL definitions for Siva 2015 and Yaremko 2022 were used. (2): fV20-perf. Alternate FL definitions for Farr 2019 and Lucia 2023 were used. (3): fMLD-vent. Alternate FL definitions for Siva 2015 and Yaremko 2022 were used. (4): fMLD-perf. Alternate FL definitions for Lucia 2023 and Farr 2019 were used.
