# Supplementary material for: Incorporation of Functional Lung Imaging Into Radiation Therapy Planning in Patients With Lung Cancer: A Systematic Review and Meta-Analysis
Source: Int J Radiat Oncol Biol Phys. Author manuscript; Available in PMC 2024 Nov 21. (PMC11580018; doi:10.1016/j.ijrobp.2024.04.001)
Supplement: Sup5 [file NIHMS2033239-supplement-Sup5.pdf]

Supplementary Table A: Comparison of SPECT Functional Lung Imaging and other Imaging Modalities

**Study**	**N**	**Cancer Type**	**Modalities**	**Results**
Bahig et al., 2017[7]	25	17 NSCLC<br>1 SCLC<br>7 Other	DECT Q<br>SPECT Q	r = 0.89, $p < 0.00001$
Castillo et al., 2012[8]	10	9 NSCLC<br>1 SCLC	CT V (HU)<br>SPECT Q	Significance not reported<br><br>*Perfusion-defect regions*<br>DSC = 0.78
Castillo et al., 2020[36]	15	All NSCLC	CT V (Jacobian)<br>CT V (HU)<br>SPECT V	SPECT VS Jacobian: Spearman r_s = 0.82, $p < 0.001$<br>SPECT VS HU: Spearman r_s = 0.49, $p < 0.001$
Castillo et al., 2021[20]	30	15 PE<br>15 Lung cancer	CT Q<br>SPECT Q	*Lung cancer*<br>Median r_s = 0.57 [95% CI = 0.45-0.71], $p < 0.00001$
Cazoulat et al., 2021[24]	6	All lung cancer	CT V (Jacobian)<br>CT V (HU)<br>CT V (Stress)<br>SPECT V	SPECT VS Jacobian: Spearman r_s = 0.39 ± 0.13; DSC = 0.44 ± 0.09<br>SPECT VS HU: Spearman r_s = 0.37 ± 0.18; DSC = 0.48 ± 0.13<br>SPECT VS Stress: Spearman r_s = 0.59 ± 0.13; DSC = 0.58 ± 0.11<br><br>Stress was significantly better than Jacobian and HU ($p < 0.001$)
Forghani et al., 2021[9]	67	All lung cancer	SPECT V<br>SPECT Q	r_s = 0.64 ± 0.19<br><br>*High-functional zone*<br>DSC = 0.54 ± 0.14
Hegi-Johnson et al., 2017[10]	11	All lung cancer	CT V (Jacobian)<br>CT V (HU with DIR)<br>CT V (HU without DIR)<br>SPECT V<br>SPECT Q	*Non-perfusion defect regions*<br>SPECT V VS Jacobian: DSC = 0.54 ± 0.13<br>SPECT V VS HU with DIR: DSC = 0.68 ± 0.54<br>SPECT V VS HU without DIR: DSC = 0.69 ± 0.08<br>SPECT V VS SPECT Q: DSC = 0.81 ± 0.05<br>SPECT Q VS Jacobian: DSC = 0.6 ± 0.14<br>SPECT Q VS HU with DIR: DSC = 0.74 ± 0.14<br>SPECT Q VS HU without DIR: DSC = 0.76 ± 0.07<br><br>*Perfusion-defect regions*<br>SPECT V VS Jacobian: DSC = 0.44 ± 0.17<br>SPECT V VS HU with DIR: DSC = 0.33 ± 0.15<br>SPECT V VS HU without DIR: DSC = 0.39 ± 0.18<br>SPECT V VS SPECT Q: DSC = 0.67 ± 0.15<br>SPECT Q VS Jacobian: 0.43 ± 0.14<br>SPECT Q VS HU with DIR: 0.35 ± 0.15<br>SPECT Q VS HU without DIR: 0.41 ± 0.2<br><br>*Whole Lung*<br>SPECT V VS Jacobian: r_s = -0.02 ± 0.11<br>SPECT V VS HU with DIR: r_s = 0.18 ± 0.1<br>SPECT V VS HU without DIR: r_s = 0.26 ±0.18<br>SPECT Q VS Jacobian: r_s = 0.03 ± 0.09<br>SPECT Q VS HU with DIR: r_s = 0.24 ± 0.12<br>SPECT Q VS HU without DIR: r_s = 0.24 ± 0.25<br>SPECT V VS SPECT Q: r_s = 0.66 ± 0.19

				No correlation was found between algorithm performance and time delay between SPECT and CT acquisition
Kipritidis et al., 2019[25]	21	All lung cancer	CT V (various)<br>SPECT V	r_s = 0.73 for the best algorithm
Lapointe et al., 2017[11]	5	All lung cancer	SPECT Q<br>DECT Q	Differential function per lobe: r = 0.91
Liu et al., 2022[26]	28	Esophagus<br>Lung	CT V (Deep Learning)<br>CT V (HU)<br>CT V (Jacobian)<br>SPECT V	SPECT V VS HU: r_s = 0.02 ± 0.1; DSC = 0.34 ± 0.04<br>SPECT V VS Jacobian: r_s = 0.02 ± 0.09; DSC = 0.34 ± 0.03<br>SPECT V VS Deep Learning: r_s = 0.65 ± 0.13/0.15; DSC = 0.59 ± 0.08/0.58 ± 0.09
Nakajima et al., 2020[12]	60	47 NSCLC<br>13 SCLC	CT V (HU)<br>SPECT Q	*Dose-function metrics compared*<br>fMLD: 0.95, p < 0.001<br>fV5: 0.97, p = 0.002<br>fV10: 0.96, p < 0.001<br>fV20: 0.95, p < 0.001<br>fV30: 0.94, p < 0.001<br>fV40: 0.94, p < 0.001
Nyeng et al., 2021[13]	30	All NSCLC	SPECT Q<br>CT V (Jacobian)	*Cutoffs based on best ROC for toxicity prediction*<br>SPECT Q VS CT: Overlap fraction median: 0.48; Intersection median: 0.09<br>SPECT V VS CT: Overlap fraction median 0.38; Intersection median: 0.13<br><br>No significant difference between comparisons to both SPECT modalities<br><br>*Cutoffs based on 1/3-volume*<br>SPECT Q VS CT: Overlap fraction median: 0.43; Intersection median: 0.12<br>SPECT V VS CT: Overlap fraction median: 0.38; Intersection median: 0.13<br>SPECT V VS SPECT Q: Overlap fraction median: 0.68; Intersection median: 0.2
Ren et al., 2021[15]	42	10 Lung cancer<br>32 Other	SPECT Q<br>CT Q	DSC: 0.8120 ± 0.0789 (functional lung)<br>R = 0.6534 ± 0.1432
Ren et al., 2022[16]	170	33 Lung cancer<br>137 other	SPECT Q<br>CT Q	DSC: 0.8112 ± 0.0484 (functional lung)<br>Voxel-wise correlation 0.8142 ± 0.0669
Pinkham et al., 2019[27]	11	All thoracic RT patients	Xe-CT<br>SPECT V	Summary statistics not reported. Only three patients had a statistically significant p-value on either a Pearson or Spearman coefficient correlation
Porter et al., 2021[14]	32	All lung cancer	SPECT Q<br>CT Q	*Pre-RT*<br>r = 0.71 [95% CI = 0.66-0.75]<br><br>*Post-RT*<br>r = 0.71 [95% CI = 0.66-0.76]<br><br>No significant performance difference found between pre- and post-RT groups
Tian et al., 2019[28]	50	20 Lung<br>30 Esophageal	CT V (Jacobian)<br>CT V (HU)<br>CT V (PRO)<br>CT V (AVG)<br>SPECT V	SPECT V VS HU: DSC = 0.4811, p ≤ 0.002<br>SPECT V VS Jac: DSC = 0.2776, p ≤ 0.002<br>SPECT V VS PRO: DSC = 0.5795, p ≤ 0.002<br>SPECT V VS AVG: DSC = 0.5932, p ≤ 0.002
Yamamoto et al., 2014[29]	8	14 Lung<br>4 Other	CT V (Jacobian)<br>CT V (HU)<br>SPECT V	SPECT V VS HU: DSC = 0.39 ± 0.11<br>SPECT V VS Jac: DSC = 0.36 ± 0.13
Yamamoto et al., 2013[30]	9	All lung cancer	CT V (Jacobian, anatomic)<br>CT V (Jacobian, phase)	Significance not reported.<br><br>SPECT V VS Jac (anatomic): DSC = 0.367

			SPECT V	SPECT V VS Jac (phase): DSC = 0.327

N: Study size

r_s: Spearman correlation coefficient

DSC: Dice similarity coefficient.
