# Supplementary material for: Incorporation of Functional Lung Imaging Into Radiation Therapy Planning in Patients With Lung Cancer: A Systematic Review and Meta-Analysis
Source: Int J Radiat Oncol Biol Phys. Author manuscript; Available in PMC 2024 Nov 21. (PMC11580018; doi:10.1016/j.ijrobp.2024.04.001)
Supplement: Sup6 [file NIHMS2033239-supplement-Sup6.docx]

| ***Supplementary Table A: Comparison of SPECT Functional Lung Imaging and other Imaging Modalities*** | | | | |
| --- | --- | --- | --- | --- |
| **Study** | **N** | **Cancer Type** | **Modalities** | **Results** |
| Bahig et al., 2017^7^ | 25 | 17 NSCLC  1 SCLC  7 Other | DECT Q  SPECT Q | r = 0.89, p < 0.00001 |
| Castillo et al., 2012^8^ | 10 | 9 NSCLC  1 SCLC | CT V (HU)  SPECT Q | Significance not reported  *Perfusion-defect regions*  DSC = 0.78 |
| Castillo et al., 2020^36^ | 15 | All NSCLC | CT V (Jacobian)  CT V (HU)  SPECT V | SPECT VS Jacobian: Spearman r_s_ = 0.82, p < 0.001  SPECT VS HU: Spearman r_s_ = 0.49, p < 0.001 |
| Castillo et al., 2021^20^ | 30 | 15 PE  15 Lung cancer | CT Q  SPECT Q | *Lung cancer*  Median r_s_ = 0.57 [95% CI = 0.45-0.71], p < 0.00001 |
| Cazoulat et al., 2021^24^ | 6 | All lung cancer | CT V (Jacobian)  CT V (HU)  CT V (Stress)  SPECT V | SPECT VS Jacobian: Spearman r_s_ = 0.39 + 0.13; DSC = 0.44 + 0.09  SPECT VS HU: Spearman r_s_ = 0.37 + 0.18; DSC = 0.48 + 0.13  SPECT VS Stress: Spearman r_s_ = 0.59 + 0.13; DSC = 0.58 + 0.11  Stress was significantly better than Jacobian and HU (p < 0.001) |
| Forghani et al., 2021^9^ | 67 | All lung cancer | SPECT V  SPECT Q | r_s_ = 0.64 + 0.19  *High-functional zone*  DSC = 0.54 + 0.14 |
| Hegi-Johnson et al., 2017^10^ | 11 | All lung cancer | CT V (Jacobian)  CT V (HU with DIR)  CT V (HU without DIR)  SPECT V  SPECT Q | *Non-perfusion defect regions*  SPECT V VS Jacobian: DSC = 0.54 + 0.13  SPECT V VS HU with DIR: DSC = 0.68 + 0.54  SPECT V VS HU without DIR: DSC = 0.69 + 0.08  SPECT V VS SPECT Q: DSC = 0.81 + 0.05  SPECT Q VS Jacobian: DSC = 0.6 + 0.14  SPECT Q VS HU with DIR: DSC = 0.74 + 0.14  SPECT Q VS HU without DIR: DSC = 0.76 + 0.07  *Perfusion-defect regions*  SPECT V VS Jacobian: DSC = 0.44 + 0.17  SPECT V VS HU with DIR: DSC = 0.33 + 0.15  SPECT V VS HU without DIR: DSC = 0.39 + 0.18  SPECT V VS SPECT Q: DSC = 0.67 + 0.15  SPECT Q VS Jacobian: 0.43 + 0.14  SPECT Q VS HU with DIR: 0.35 + 0.15  SPECT Q VS HU without DIR: 0.41 + 0.2  *Whole Lung*  SPECT V VS Jacobian: r_s_ = -0.02 + 0.11  SPECT V VS HU with DIR: r_s_ = 0.18 + 0.1  SPECT V VS HU without DIR: r_s_ = 0.26 +0.18  SPECT Q VS Jacobian: r_s_ = 0.03 + 0.09  SPECT Q VS HU with DIR: r_s_ = 0.24 + 0.12  SPECT Q VS HU without DIR: r_s_ = 0.24 + 0.25  SPECT V VS SPECT Q: r_s_ = 0.66 + 0.19  No correlation was found between algorithm performance and time delay between SPECT and CT acquisition |
| Kipritidis et al., 2019^25^ | 21 | All lung cancer | CT V (various)  SPECT V | r_s_ = 0.73 for the best algorithm |
| Lapointe et al., 2017^11^ | 5 | All lung cancer | SPECT Q  DECT Q | Differential function per lobe: r = 0.91 |
| Liu et al., 2022^26^ | 28 | Esophagus  Lung | CT V (Deep Learning)  CT V (HU)  CT V (Jacobian)  SPECT V | SPECT V VS HU: r_s_ = 0.02 + 0.1; DSC = 0.34 + 0.04  SPECT V VS Jacobian: r_s_ = 0.02 + 0.09; DSC = 0.34 + 0.03  SPECT V VS Deep Learning: r_s_ = 0.65 + 0.13/0.15; DSC = 0.59 + 0.08/0.58 + 0.09 |
| Nakajima et al., 2020^12^ | 60 | 47 NSCLC  13 SCLC | CT V (HU)  SPECT Q | *Dose-function metrics compared*  fMLD: 0.95, p < 0.001  fV5: 0.97, p = 0.002  fV10: 0.96, p < 0.001  fV20: 0.95, p < 0.001  fV30: 0.94, p < 0.001  fV40: 0.94, p < 0.001 |
| Nyeng et al., 2021^13^ | 30 | All NSCLC | SPECT Q  CT V (Jacobian) | *Cutoffs based on best ROC for toxicity prediction*  SPECT Q VS CT: Overlap fraction median: 0.48; Intersection median: 0.09  SPECT V VS CT: Overlap fraction median 0.38; Intersection median: 0.13  No significant difference between comparisons to both SPECT modalities  *Cutoffs based on 1/3-volume*  SPECT Q VS CT: Overlap fraction median: 0.43; Intersection median: 0.12  SPECT V VS CT: Overlap fraction median: 0.38; Intersection median: 0.13  SPECT V VS SPECT Q: Overlap fraction median: 0.68; Intersection median: 0.2 |
| Ren et al., 2021^15^ | 42 | 10 Lung cancer  32 Other | SPECT Q  CT Q | DSC: 0.8120 + 0.0789 (functional lung)  R = 0.6534 + 0.1432 |
| Ren et al., 2022^16^ | 170 | 33 Lung cancer  137 other | SPECT Q  CT Q | DSC: 0.8112 + 0.0484 (functional lung)  Voxel-wise correlation 0.8142 + 0.0669 |
| Pinkham et al., 2019^27^ | 11 | All thoracic RT patients | Xe-CT  SPECT V | Summary statistics not reported. Only three patients had a statistically significant p-value on either a Pearson or Spearman coefficient correlation |
| Porter et al., 2021^14^ | 32 | All lung cancer | SPECT Q  CT Q | *Pre-RT*  r = 0.71 [95% CI = 0.66-0.75]  *Post-RT*  r = 0.71 [95% CI = 0.66-0.76]  No significant performance difference found between pre- and post-RT groups |
| Tian et al., 2019^28^ | 50 | 20 Lung  30 Esophageal | CT V (Jacobian)  CT V (HU)  CT V (PRO)  CT V (AVG)  SPECT V | SPECT V VS HU: DSC = 0.4811, p < 0.002  SPECT V VS Jac: DSC = 0.2776, p < 0.002  SPECT V VS PRO: DSC = 0.5795, p < 0.002  SPECT V VS AVG: DSC = 0.5932, p < 0.002 |
| Yamamoto et al., 2014^29^ | 8 | 14 Lung  4 Other | CT V (Jacobian)  CT V (HU)  SPECT V | SPECT V VS HU: DSC = 0.39 + 0.11  SPECT V VS Jac: DSC = 0.36 + 0.13 |
| Yamamoto et al., 2013^30^ | 9 | All lung cancer | CT V (Jacobian, anatomic)  CT V (Jacobian, phase)  SPECT V | Significance not reported.  SPECT V VS Jac (anatomic): DSC = 0.367  SPECT V VS Jac (phase): DSC = 0.327 |

N: Study size

r_s_: Spearman correlation coefficient

DSC: Dice similarity coefficient.
