# Supplementary material for: Incorporation of Functional Lung Imaging Into Radiation Therapy Planning in Patients With Lung Cancer: A Systematic Review and Meta-Analysis
Source: Int J Radiat Oncol Biol Phys. Author manuscript; Available in PMC 2024 Nov 21. (PMC11580018; doi:10.1016/j.ijrobp.2024.04.001)
Supplement: Sup7 [file NIHMS2033239-supplement-Sup7.pdf]

Supplementary Table B

Study	FLI Modality	Definition of Functional Lung
Agrawal et al., 2012[137]	SPECT Q	Visual thresholding
Allen et al., 2011[66]	He-MRI V	Whole Volume Used
Bin et al., 2021[143]	CT V (HU)	Weighting
Bahig et al., 2017[7]	SPECT Q<br>DECT Q	SPECT Q: weighted based on SPECT counts<br>DECT Q: weighted based on iodine content<br><br>Both then manually thresholded into 6 functional subvolumes
Bucknell et al., 2023[82]	Ga-PET V/Q	70th percentile thresholding, followed by manual review from a nuclear medicine physician
Cai et al., 2011[80]	He-MRI V	Thresholded at 30% of the maximum signal
Castillo et al., 2012[8]	CT V (HU)<br>SPECT Q	Auto-thresholding via percentile (unspecified), followed by manual editing
Cazoulat et al., 2021[24]	CT V (Jac)<br>CT V (HU)<br>CT V (Stress)<br>SPECT V	Thresholded at 25% and 75% of max.
Christian et al., 2005[83]	SPECT Q	Visual thresholding into two groups; weighting
Das et al., 2004[31]	SPECT Q	Weighting
Defraene et al., 2019[67]	CT (Direct Threshold)	Thresholding of changes in HU values
Dhami et al., 2017[84]	SPECT Q	Thresholding: thresholding from 5% to 95% of max, in 5% increments
Ding et al., 2018[73]	SPECT Q	Thresholding: thresholding from 10% to 60% of max, in 10% increments<br><br>Weighting
Ding et al., 2022[85]	Xe-MRI V	Thresholded into four groups (unspecified)

Doi et al., 2017[151]	CT (Direct Thresholding)	Direct thresholding at -860 HU
Dougherty et al., 2021[86]	CT V (HU)	15% thresholding, followed by other unspecified segmentation
Farr et al., 2019[87]	SPECT Q	Thresholding: from 20% to 80% of maximum perfusion, in steps of 10%
Farr et al., 2015[121]	SPECT Q	Thresholding: from 20% to 80% of maximum perfusion, in steps of 10%, followed by weighting
Farr et al., 2015[72]	SPECT Q	Thresholding: from 20% to 80% of maximum perfusion, in steps of 20%
Faught et al., 2017[89]	CT V (HU)	Thresholding: functional voxels are no more than 15% less ventilated than the average ventilation of the entire lung, were it to be homogeneous
Faught et al., 2018[88]	CT V (HU)	Thresholding: functional voxels are no more than 15% less ventilated than the average ventilation of the entire lung, were it to be homogeneous
Faught et al., 2017[90]	CT V (HU)	Thresholding: from 5th to 95th percentile<br><br>Nonlinear weighting: using a sigmoidal function.
Feng et al., 2021[39]	CT V (Jacobian with density)	Thresholding Jacobian ventilation at 30% and 60% of max<br><br>Thresholding density at -700HU and -850 HU<br><br>Both factors were taken into account in the same functional plan
Follacchio et al., 2020[76]	SPECT Q	Thresholded at 60% of maximum
Forghani et al., 2021[9]	SPECT Q<br>SPECT V	Low function: 0-25th percentile<br>High function: 75th percentile onwards
Greco et al., 2022[91]	SPECT Q	Thresholding: Thresholded at 70% and 40% to 70% of max
Grover et al., 2022[55]	CT V (Deep Learning)<br>Ga-PET V	Thresholded into three equal volumes, for both modalities
Guerrero et al., 2005[47]	CT V (HU)	Automated segmentation with subsequent editing
Hardcastle et al., 2015[59]	Ga-PET Q	Thresholding: 70% of max, with sub-thresholds within based on isodose lines
Hegi-Johnson et al., 2017[10]	SPECT V<br>SPECT Q<br>CT V (Jacobian) CT V (HU no DIR)<br>CT V (HU with DIR)	SPECT: thresholded at 50% of the 90th percentile within the ROI<br><br>CT: increments of 5% to 95%, in steps of 5%, of 90th percentile ventilation within the ROI; selected at 20% for HU with DIR, 30% for Jacobian, and 70% for HU without DIR

Hoover et al., 2013[144]	SPECT V/Q	Weighting
Hodge et al., 2010[135]	He-MRI V	Automated Threshold
Huang et al., 2018[92]	CT V (Jacobian)	Thresholding, unspecified
Huang et al., 2013[71]	CT V (breath change)	Thresholded at 20%, 30% and 40% of maximum
Huang et al., 2021[140]	CT V (HU)	Weighting
Huang et al., 2023[136]	CT V (Xenon-enhanced)	Automatic segmentation of Xe-ehnanced areas at > 15 HU
Ieko et al., 2020[93]	CT V (HU)	Thresholding at 20th percentile
Ireland et al., 2007[139]	He-MRI V	All He-defined lung
Ireland et al., 2010[138]	He-MRI V	All He-defined lung
Iqbal et al., 2023[81]	CT V (Density)	Density change-based algorithm, with weighting or thresholding at 33% and 66% of maximum
Jafari et al., 2019[56]	CT V	Weighting
Kadoya et al., 2015[120]	CT V (Jacobian)	Threshold: 90th percentile
Kanai et al., 2018[96]	CT V (HU)	Thresholding: different thresholds, from 5th to 95th percentiles, in increments of 5 percentiles<br>Weighting
Kazemzadeh et al., 2018[54]	CT V: Large airways	CT-based mapping of the bronchial tree
Kida et al., 2016[77]	CT V<br>SPECT V	Thresholded at the mean function, plus/minus the standard deviation of function
Kimura et al., 2015[147]	CT (Direct threshold)<br>SPECT Q	CT: Direct thresholding of HU values at -860 and -910 HU<br><br>SPECT Q: Percentile thresholds at 10, 30, 50, and 70th percentiles
Kimura et al., 2012[69]	CT (Direct threshold)	Threshold: -860 HU

Kipritidis et al., 2019[25]	CT V (various)<br>SPECT V	Threshold at 25th, 50th, 75th percentile
Kipritidis et al., 2015[45]	CT V (Jacobian)<br>Ga-PET V	Weighting
Kipritidis et al., 2015[40]	CT V (HU)<br>Ga-PET V	PET: Thresholded at 10-30% maximum<br><br>CT: Threshold algorithm, unspecified
Kipritidis et al., 2013[34]	CT V (HU)<br>CT V (Jacobian)<br>Ga-PET V	Threshold at 21-100th percentile range
Kocak et al., 2007[141]	SPECT Q	Weighted
Lan et al., 2016[50]	CT V (Density change)	Weighted<br><br>Thresholded: 20%, 40%, 60%, 80% of max
Lapointe et al., 2017[11]	DECT Q<br>SPECT Q	Threshold -250 HU for DECT<br>SPECT not specified
Latifi et al., 2013[41]	CT V (change in volume)<br>CT V (Jacobian)<br>CT V (HU)	Threshold: 20% and 80% of maximum
Lavrenkov et al., 2007[94]	SPECT Q	Threshold: 60% of maximum
Lavrenkov et al., 2009[95]	SPECT Q	Threshold: 60% of maximum
Lee et al., 2017[21]	SPECT Q	Threshold into seven equidistant bins; 70% of max threshold used for dose-function parameters
Lee et al., 2018[79]	SPECT Q	Threshold into seven equidistant bins; 70% of max threshold used for dose-function parameters
Le Roux et al., 2015[132]	PET V/Q	Independent review
Le Roux et al., 2017[60]	PET V/Q	Thresholded at 15% of maximum
Li et al., 2023[97]	CT V (Jac)	Threshold: top 10, 20, 30, 40, and 50% of maximum each (planning)<br>Weighting (dose-function parameter calculation)
Li et al., 2022[130]	CT Q	Threshold at 0.3
Li et al., 2022[58]	Ga-PET V/Q	Thresholded based on deviation from 0 value, cut-off ranging from 0.05 to 0.4 for log(V) or log(Q)

Lind et al., 2002[142]	SPECT Q	Weighting
Liu et al., 2022[26]	CT V (Deep Learning)<br>CT V (HU)<br>CT V (Jac)<br>SPECT V	Thresholded into equidistant thirds.
Lucia et al., 2023[116]	Ga-PET Q	Minimal volume containing top 50, 70, and 90% of total activity each.
Mathew et al., 2012[43]	He-MRI V<br>CT V (HU)	Automated
Matrosic et al., 2021[68]	CT PRM	Normal VS parenchymal VS small airway VS emphysema
Matuszak et al., 2016[146]	SPECT Q	Weighting
McGuire et al., 2006[108]	SPECT Q	Threshold into four regions, with subsequent weighting
McGuire et al., 2009[127]	SPECT Q	Threshold into four regions, with subsequent weighting
McIntosh et al., 2021[63]	Ga-PET V/Q	Threshold at 5% to 70% of peak intensity, in increments of 5
Meng et al., 2014[74]	SPECT Q<br>SPECT V	Thresholds: top 30%, top 60%
Miften et al., 2004[134]	SPECT Q	Manual thresholding
Miller et al., 2023[148]	CT V (HU-based)	Threshold: 15% of maximum
Mounessi et al., 2020[114]	SPECT Q	Threshold: Top 30% of maximum
Munawar et al., 2010[117]	SPECT V	Thresholded at 50% or 70% of maximum<br><br>Weighting
Nakajima et al., 2020[12]	SPECT Q<br>CT V (HU)	Weighting
Nyeng et al., 2021[13]	CT V (Jacobian)<br>SPECT Q	SPECT: threshold various<br><br>CT V: threshold various
Nyeng et al., 2011[42]	CT V (Jacobian)	Thresholded at Det(J) > 1

O'Reilly et al., 2020[105]	CT V (Jacobian)	Threshold at top 6%, 45%, and 60% of max
Otsuka et al., 2018[101]	CT V (Jacobian)	Threshold at percentile regions from 0 to 100, in increments of 10
Owen et al., 2021[106]	SPECT V/Q	Percentile thresholds from 10 to 90, in increments of 10;
Patton et al., 2018[98]	CT V (Jacobian)	Threshold at Jacobian = 1.1
Porter et al., 2021[14]	SPECT Q<br>CT Q (Deep Learning)	Threshold at 50th percentile
Rankine et al., 2018[65]	Xe-MRI V	Threshold into equal thirds
Ren et al., 2021[15]	SPECT Q<br>CT Q	Threshold at 0.66
Ren et al., 2021[16]	SPECT Q<br>CT Q	Threshold at 0.66
Seppenwoolde et al., 2002[149]	SPECT Q	Weighting
Sharifi et al., 2019[78]	CT V (Jacobian)<br>CT V (Volume)	Threshold at 95% of max and weighting
Shioyama et al., 2007[110]	SPECT Q	Thresholding at 50th and 90th percentiles
Siva et al., 2015[111]	Ga-PET V/Q	Perfusion 70th percentile<br>Ventilation 70th and 50th percentile
Siva et al., 2016[112]	Ga-PET Q	Top 70% of voxels
St-Hilaire et al., 2011[150]	SPECT Q	Weighting
Suga et al., 2004[17]	SPECT V	Visual delineations
Thomas et al., 2019[102]	SPECT Q	Threshold at 70% maximum
Thomas et al., 2022[126]	SPECT Q	Radiomics, thresholding and weighting separately each
Tian et al., 2019[28]	CT V (Jacobian)<br>CT V (HU)	Segment into 3 equal volumes

	CT V (PRO)<br>CT V (AVG)<br>SPECT V	
Vicente et al., 2020[53]	CT V (HU and Jacobian hybrid)<br><br>CT V (Airway)	Thresholding at average of pre-treatment scan<br><br>Airway: functional if it has <5% probability of collapse
Vicente et al., 2022[57]	CT V (HU and Jacobian hybrid)<br><br>CT V (Airway)	Thresholding at average of pre-treatment scan<br><br>Airway: functional if it has <5% probability of collapse
Vinogradskiy et al., 2011[75]	CT V (HU)	Threshold at 50th percentile
Vinogradskiy et al., 2013[147]	CT V (HU)	Weighting
Vinogradskiy et al., 2016[129]	CT V (HU)	Weighting<br>Threshold: 20% of maximum
Vinogradskiy et al., 2022[119]	CT V (HU)	Threshold: 15% of maximum
Wang et al., 2013[113]	SPECT Q	Threshold: 30% of maximum
Wang et al., 2014[115]	CT V (Jacobian)	Threshold: Top 30%
Wang et al., 2011[99]	SPECT Q	Thresholding at 30% of maximum, followed by weighting
Wang et al., 2012[104]	SPECT Q	Threshold at 30% maximum, followed by weighting
Wang et al., 2012[123]	SPECT Q	Threshold at 30% maximum, followed by weighting
Waxweiler et al., 2015[118]	CT V (HU)	maximum 15% reduction per lung third, weighted
Weller et al., 2019[100]	SPECT Q	Thresholding at 20% of maximum
Woodruff et al., 2017[49]	CT V (In-house software)	Low function thresholded at lowest quartile of lung function
Xiao et al., 2017[125]	SPECT V<br>SPECT Q	Thresholding at 30% of max

Xiao et al., 2018[103]	SPECT Q	Thresholding at 30% of max
Yamamoto et al., 2016[145]	CT V (HU)	Weighted
Yamamoto et al., 2011[109]	CT V (Jacobian)	Thresholding into three equal volumes
Yamamoto et al., 2013[29]	CT V (Jacobian, anatomic)<br>CT V (Jacobian, phase)<br>SPECT V	Thresholded at 33% of total volume
Yamamoto et al., 2014[30]	CT V (Jacobian)<br>CT V (HU)<br>SPECT V	CT: Thresholding at 25% of total volume<br>SPECT: Threshold at mean density of background noise, plus two standard deviations
Yamamoto et al., 2012[32]	CT V (Jacobian)	Thresholded into three regions of equivalent volume
Yamamoto et al., 2018[145]	CT V (Elastic)	Weighting
Yaremko et al., 2022[133]	He-MRI V	Automated thresholding
Yaremko et al., 2007[107]	CT V (HU)	Thresholding at 90th percentile
Yuan et al., 2011[22]	SPECT V<br>SPECT Q	Annotation by nuclear medicine physician
Yin et al., 2009[131]	SPECT Q	Visual thresholding and thresholding at 30% of maximum
Yin et al., 2009[19]	SPECT Q	Thresholding at 10% of maximum
Yin et al., 2010[18]	SPECT Q	Thresholding at 10 to 90% of maximum, in 10% increments

Supplementary B: Definition of functional lung used in each paper, when provided. Some studies provided rationales for their choice(s) of method, in which case this is detailed in the final column.
