# Supplementary material for: Incorporation of Functional Lung Imaging Into Radiation Therapy Planning in Patients With Lung Cancer: A Systematic Review and Meta-Analysis
Source: Int J Radiat Oncol Biol Phys. Author manuscript; Available in PMC 2024 Nov 21. (PMC11580018; doi:10.1016/j.ijrobp.2024.04.001)
Supplement: Sup8 [file NIHMS2033239-supplement-Sup8.docx]

**Supplementary Table B**

| **Study** | **FLI Modality** | **Definition of Functional Lung** |
| --- | --- | --- |
| Agrawal et al., 2012^137^ | SPECT Q | Visual thresholding |
| Allen et al., 2011^67^ | He-MRI V | Whole Volume Used |
| Bin et al., 2021^143^ | CT V (HU) | Weighting |
| Bahig et al., 2017^7^ | SPECT Q  DECT Q | SPECT Q: weighted based on SPECT counts  DECT Q: weighted based on iodine content    Both then manually thresholded into 6 functional subvolumes |
| Bucknell et al., 2023^82^ | Ga-PET V/Q | 70^th^ percentile thresholding, followed by manual review from a nuclear medicine physician |
| Cai et al., 2011^80^ | He-MRI V | Thresholded at 30% of the maximum signal |
| Castillo et al., 2012^8^ | CT V (HU)  SPECT Q | Auto-thresholding via percentile (unspecified), followed by manual editing |
| Cazoulat et al., 2021^24^ | CT V (Jac)  CT V (HU)  CT V (Stress)  SPECT V | Thresholded at 25% and 75% of max. |
| Christian et al., 2005^83^ | SPECT Q | Visual thresholding into two groups; weighting |
| Das et al., 2004^31^ | SPECT Q | Weighting |
| Defraene et al., 2019^67^ | CT (Direct Threshold) | Thresholding of changes in HU values |
| Dhami et al., 2017^84^ | SPECT Q | Thresholding: thresholding from 5% to 95% of max, in 5% increments |
| Ding et al., 2018^73^ | SPECT Q | Thresholding: thresholding from 10% to 60% of max, in 10% increments  Weighting |
| Ding et al., 2022^85^ | Xe-MRI V | Thresholded into four groups (unspecified) |
| Doi et al., 2017^151^ | CT (Direct Thresholding) | Direct thresholding at -860 HU |
| Dougherty et al., 2021^86^ | CT V (HU) | 15% thresholding, followed by other unspecified segmentation |
| Farr et al., 2019^87^ | SPECT Q | Thresholding: from 20% to 80% of maximum perfusion, in steps of 10% |
| Farr et al., 2015^121^ | SPECT Q | Thresholding: from 20% to 80% of maximum perfusion, in steps of 10%, followed by weighting |
| Farr et al., 2015^72^ | SPECT Q | Thresholding: from 20% to 80% of maximum perfusion, in steps of 20% |
| Faught et al., 2017^89^ | CT V (HU) | Thresholding: functional voxels are no more than 15% less ventilated than the average ventilation of the entire lung, were it to be homogeneous |
| Faught et al., 2018^88^ | CT V (HU) | Thresholding: functional voxels are no more than 15% less ventilated than the average ventilation of the entire lung, were it to be homogeneous |
| Faught et al., 2017^90^ | CT V (HU) | Thresholding: from 5^th^ to 95^th^ percentile    Nonlinear weighting: using a sigmoidal function. |
| Feng et al., 2021^39^ | CT V (Jacobian with density) | Thresholding Jacobian ventilation at 30% and 60% of max    Thresholding density at -700HU and -850 HU    Both factors were taken into account in the same functional plan |
| Follacchio et al., 2020^76^ | SPECT Q | Thresholded at 60% of maximum |
| Forghani et al., 2021^9^ | SPECT Q  SPECT V | Low function: 0-25^th^ percentile  High function: 75^th^ percentile onwards |
| Greco et al., 2022^91^ | SPECT Q | Thresholding: Thresholded at 70% and 40% to 70% of max |
| Grover et al., 2022^55^ | CT V (Deep Learning)  Ga-PET V | Thresholded into three equal volumes, for both modalities |
| Guerrero et al., 2005^48^ | CT V (HU) | Automated segmentation with subsequent editing |
| Hardcastle et al., 2015^59^ | Ga-PET Q | Thresholding: 70% of max, with sub-thresholds within based on isodose lines |
| Hegi-Johnson et al., 2017^10^ | SPECT V  SPECT Q  CT V (Jacobian) CT V (HU no  DIR)  CT V (HU with DIR) | SPECT: thresholded at 50% of the 90^th^ percentile within the ROI    CT: increments of 5% to 95%, in steps of 5%, of 90^th^ percentile ventilation within the ROI; selected at 20% for HU with DIR, 30% for Jacobian, and 70% for HU without DIR |
| Hoover et al., 2013^144^ | SPECT V/Q | Weighting |
| Hodge et al., 2010^135^ | He-MRI V | Automated Threshold |
| Huang et al., 2018^92^ | CT V (Jacobian) | Thresholding, unspecified |
| Huang et al., 2013^71^ | CT V (breath change) | Thresholded at 20%, 30% and 40% of maximum |
| Huang et al., 2021^140^ | CT V (HU) | Weighting |
| Huang et al., 2023^136^ | CT V (Xenon-enhanced) | Automatic segmentation of Xe-ehnanced areas at > 15 HU |
| Ieko et al., 2020^93^ | CT V (HU) | Thresholding at 20^th^ percentile |
| Ireland et al., 2007^139^ | He-MRI V | All He-defined lung |
| Ireland et al., 2010^138^ | He-MRI V | All He-defined lung |
| Iqbal et al., 2023^81^ | CT V (Density) | Density change-based algorithm, with weighting or thresholding at 33% and 66% of maximum |
| Jafari et al., 2019^56^ | CT V | Weighting |
| Kadoya et al., 2015^120^ | CT V (Jacobian) | Threshold: 90^th^ percentile |
| Kanai et al., 2018^96^ | CT V (HU) | Thresholding: different thresholds, from 5^th^ to 95^th^ percentiles, in increments of 5 percentiles  Weighting |
| Kazemzadeh et al., 2018^54^ | CT V: Large airways | CT-based mapping of the bronchial tree |
| Kida et al., 2016^77^ | CT V  SPECT V | Thresholded at the mean function, plus/minus the standard deviation of function |
| Kimura et al., 2015^147^ | CT (Direct threshold)  SPECT Q | CT: Direct thresholding of HU values at -860 and -910 HU    SPECT Q: Percentile thresholds at 10, 30, 50, and 70^th^ percentiles |
| Kimura et al., 2012^69^ | CT (Direct threshold) | Threshold: -860 HU |
| Kipritidis et al., 2019^25^ | CT V (various)  SPECT V | Threshold at 25^th^, 50^th^, 75^th^ percentile |
| Kipritidis et al., 2015^44^ | CT V (Jacobian)  Ga-PET V | Weighting |
| Kipritidis et al., 2015^40^ | CT V (HU)  Ga-PET V | PET: Thresholded at 10-30% maximum  CT: Threshold algorithm, unspecified |
| Kipritidis et al., 2013^34^ | CT V (HU) CT V (Jacobian) Ga-PET V | Threshold at 21-100^th^ percentile range |
| Kocak et al., 2007^141^ | SPECT Q | Weighted |
| Lan et al., 2016^50^ | CT V (Density change) | Weighted    Thresholded: 20%, 40%, 60%, 80% of max |
| Lapointe et al., 2017^11^ | DECT Q  SPECT Q | Threshold -250 HU for DECT  SPECT not specified |
| Latifi et al., 2013^41^ | CT V (change in volume)  CT V (Jacobian)  CT V (HU) | Threshold: 20% and 80% of maximum |
| Lavrenkov et al., 2007^94^ | SPECT Q | Threshold: 60% of maximum |
| Lavrenkov et al., 2009^95^ | SPECT Q | Threshold: 60% of maximum |
| Lee et al., 2017^21^ | SPECT Q | Threshold into seven equidistant bins; 70% of max threshold used for dose-function parameters |
| Lee et al., 2018^79^ | SPECT Q | Threshold into seven equidistant bins; 70% of max threshold used for dose-function parameters |
| Le Roux et al., 2015^132^ | PET V/Q | Independent review |
| Le Roux et al., 2017^60^ | PET V/Q | Thresholded at 15% of maximum |
| Li et al., 2023^97^ | CT V (Jac) | Threshold: top 10, 20, 30, 40, and 50% of maximum each (planning)  Weighting (dose-function parameter calculation) |
| Li et al., 2022^130^ | CT Q | Threshold at 0.3 |
| Li et al., 2022^58^ | Ga-PET V/Q | Thresholded based on deviation from 0 value, cut-off ranging from 0.05 to 0.4 for log(V) or log(Q) |
| Lind et al., 2002^142^ | SPECT Q | Weighting |
| Liu et al., 2022^26^ | CT V (Deep Learning)  CT V (HU)  CT V (Jac)  SPECT V | Thresholded into equidistant thirds. |
| Lucia et al., 2023^116^ | Ga-PET Q | Minimal volume containing top 50, 70, and 90% of total activity each. |
| Mathew et al., 2012^46^ | He-MRI V  CT V (HU) | Automated |
| Matrosic et al., 2021^65^ | CT PRM | Normal VS parenchymal VS small airway VS emphysema |
| Matuszak et al., 2016^146^ | SPECT Q | Weighting |
| McGuire et al., 2006^108^ | SPECT Q | Threshold into four regions, with subsequent weighting |
| McGuire et al., 2009^127^ | SPECT Q | Threshold into four regions, with subsequent weighting |
| McIntosh et al., 2021^62^ | Ga-PET V/Q | Threshold at 5% to 70% of peak intensity, in increments of 5 |
| Meng et al., 2014^74^ | SPECT Q  SPECT V | Thresholds: top 30%, top 60% |
| Miften et al., 2004^134^ | SPECT Q | Manual thresholding |
| Miller et al., 2023^148^ | CT V (HU-based) | Threshold: 15% of maximum |
| Mounessi et al., 2020^114^ | SPECT Q | Threshold: Top 30% of maximum |
| Munawar et al., 2010^117^ | SPECT V | Thresholded at 50% or 70% of maximum    Weighting |
| Nakajima et al., 2020^12^ | SPECT Q  CT V (HU) | Weighting |
| Nyeng et al., 2021^13^ | CT V (Jacobian)  SPECT Q | SPECT: threshold various    CT V: threshold various |
| Nyeng et al., 2011^42^ | CT V (Jacobian) | Thresholded at Det(J) > 1 |
| O’Reilly et al., 2020^105^ | CT V (Jacobian) | Threshold at top 6%, 45%, and 60% of max |
| Otsuka et al., 2018^101^ | CT V (Jacobian) | Threshold at percentile regions from 0 to 100, in increments of 10 |
| Owen et al., 2021^106^ | SPECT V/Q | Percentile thresholds from 10 to 90, in increments of 10; |
| Patton et al., 2018^98^ | CT V (Jacobian) | Threshold at Jacobian = 1.1 |
| Porter et al., 2021^14^ | SPECT Q  CT Q (Deep Learning) | Threshold at 50^th^ percentile |
| Rankine et al., 2018^66^ | Xe-MRI V | Threshold into equal thirds |
| Ren et al., 2021^15^ | SPECT Q  CT Q | Threshold at 0.66 |
| Ren et al., 2021^16^ | SPECT Q  CT Q | Threshold at 0.66 |
| Seppenwoolde et al., 2002^149^ | SPECT Q | Weighting |
| Sharifi et al., 2019^78^ | CT V (Jacobian) CT V (Volume) | Threshold at 95% of max and weighting |
| Shioyama et al., 2007^110^ | SPECT Q | Thresholding at 50^th^ and 90^th^ percentiles |
| Siva et al., 2015^111^ | Ga-PET V/Q | Perfusion 70^th^ percentile  Ventilation 70^th^ and 50^th^ percentile |
| Siva et al., 2016^112^ | Ga-PET Q | Top 70% of voxels |
| St-Hilaire et al., 2011^150^ | SPECT Q | Weighting |
| Suga et al., 2004^17^ | SPECT V | Visual delineations |
| Thomas et al., 2019^102^ | SPECT Q | Threshold at 70% maximum |
| Thomas et al., 2022^126^ | SPECT Q | Radiomics, thresholding and weighting separately each |
| Tian et al., 2019^28^ | CT V (Jacobian)  CT V (HU)  CT V (PRO)  CT V (AVG)  SPECT V | Segment into 3 equal volumes |
| Vicente et al., 2020^53^ | CT V (HU and Jacobian  hybrid)    CT V (Airway) | Thresholding at average of pre-treatment scan  Airway: functional if it has <5% probability of collapse |
| Vicente et al., 2022^57^ | CT V (HU and Jacobian  hybrid)    CT V (Airway) | Thresholding at average of pre-treatment scan  Airway: functional if it has <5% probability of collapse |
| Vinogradskiy et al., 2011^75^ | CT V (HU) | Threshold at 50^th^ percentile |
| Vinogradskiy et al., 2013^147^ | CT V (HU) | Weighting |
| Vinogradskiy et al., 2016^129^ | CT V (HU) | Weighting  Threshold: 20% of maximum |
| Vinogradskiy et al., 2022^119^ | CT V (HU) | Threshold: 15% of maximum |
| Wang et al., 2013^113^ | SPECT Q | Threshold: 30% of maximum |
| Wang et al., 2014^115^ | CT V (Jacobian) | Threshold: Top 30% |
| Wang et al., 2011^99^ | SPECT Q | Thresholding at 30% of maximum, followed by weighting |
| Wang et al., 2012^104^ | SPECT Q | Threshold at 30% maximum, followed by weighting |
| Wang et al., 2012^123^ | SPECT Q | Threshold at 30% maximum, followed by weighting |
| Waxweiler et al., 2015^118^ | CT V (HU) | maximum 15% reduction per lung third, weighted |
| Weller et al., 2019^100^ | SPECT Q | Thresholding at 20% of maximum |
| Woodruff et al., 2017^45^ | CT V (In-house software) | Low function thresholded at lowest quartile of lung function |
| Xiao et al., 2017^125^ | SPECT V  SPECT Q | Thresholding at 30% of max |
| Xiao et al., 2018^103^ | SPECT Q | Thresholding at 30% of max |
| Yamamoto et al., 2016^145^ | CT V (HU) | Weighted |
| Yamamoto et al., 2011^109^ | CT V (Jacobian) | Thresholding into three equal volumes |
| Yamamoto et al., 2013^29^ | CT V (Jacobian, anatomic)  CT V (Jacobian, phase) SPECT V | Thresholded at 33% of total volume |
| Yamamoto et al., 2014^30^ | CT V (Jacobian)  CT V (HU)  SPECT V | CT: Thresholding at 25% of total volume  SPECT: Threshold at mean density of background noise, plus two standard deviations |
| Yamamoto et al., 2012^32^ | CT V (Jacobian) | Thresholded into three regions of equivalent volume |
| Yamamoto et al., 2018^145^ | CT V (Elastic) | Weighting |
| Yaremko et al., 2022^133^ | He-MRI V | Automated thresholding |
| Yaremko et al., 2007^107^ | CT V (HU) | Thresholding at 90^th^ percentile |
| Yuan et al., 2011^22^ | SPECT V  SPECT Q | Annotation by nuclear medicine physician |
| Yin et al., 2009^131^ | SPECT Q | Visual thresholding and thresholding at 30% of maximum |
| Yin et al., 2009^19^ | SPECT Q | Thresholding at 10% of maximum |
| Yin et al., 2010^18^ | SPECT Q | Thresholding at 10 to 90% of maximum, in 10% increments |

Supplementary B: Definition of functional lung used in each paper, when provided. Some studies provided rationales for their choice(s) of method, in which case this is detailed in the final column.
